# Formation and Structure of Hydrolytic Methylaluminoxane Activators

**DOI:** 10.1002/chem.202102463

**Published:** 2021-10-06

**Authors:** Scott Collins, Anuj Joshi, Mikko Linnolahti

**Affiliations:** ^1^ Former affiliation: Department of Chemistry University of Victoria 3800 Finnerty Road Victoria BC V8P 5C2 Canada).; ^2^ UVic Genome BC Proteomics Research Centre sup 4464 Markham St #3101 Victoria BC V8Z 5N3 Canada; ^3^ Department of Chemistry University of Eastern Finland, Joensuu Campus Yliopistokatu 7 80100 Joensuu Finland

**Keywords:** aggregation, DFT, ESI-MS, methylaluminoxane, numerical simulation

## Abstract

Methylaluminoxane (MAO) activators have sheet structures which form ion‐pairs on reaction of neutral donors such as octamethyltrisiloxane (OMTS). The ion‐pairs can be detected by electrospray ionization mass spectrometry (ESI‐MS) in polar media. The growth of these reactive precursors during hydrolysis of Me_3_Al can be monitored using ESI‐MS. Density functional theory, combined with numerical simulation of growth, indicates that this process involves rapid formation of low MW oligomers, followed by assembly of these species into low MW sheets. These can grow through further addition of low MW oligomers or by fusion into larger sheets. The mechanism of these growth processes leads to the prediction that even‐numbered sheets should be favored, and this surprising result is confirmed by ESI‐MS monitoring experiments of both activator growth and MAO aging.

## Graphical abstract

**Modelling of kinetics** using density functional theory, combined with numerical simulation, reveals that methylaluminoxane activators are sheets formed by assembly of linear oligomers, wherein the statistics and mechanism of aggregation favor formation of even‐numbered sheets.

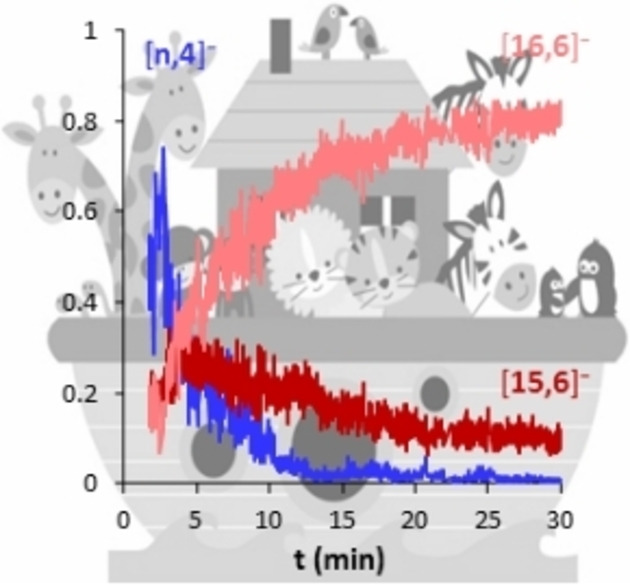

## Introduction

Hydrolytic methylaluminoxane (h‐MAO) is often used as an activator in single‐site polymerization catalysis. It serves multiple roles such as scavenging of impurities, alkylation of the catalyst precursor, and ionization to form active catalyst.^[1]^ Until quite recently, it was the only commonly used activator that possessed all of these desirable properties.^[2]^

Despite its useful properties, a large excess is required for high activity in solution^[1]^ and it is rather expensive compared to other alkylaluminoxanes as it is prepared from Me_3_Al by a controlled hydrolysis.^[3]^ Further, excess Me_3_Al present in all samples of h‐MAO can have deleterious effects on catalyst activity.^[4]^

The commercial production of h‐MAO is achieved in a continuous process involving intimate mixing of water and a 3–5 fold excess of Me_3_Al in dilute toluene suspension. The process is conducted isothermally below room temperature to provide a mixture of h‐MAO and unreacted Me_3_Al with very little gel formation.^[3]^ Gel formation involves a local excess of water over R_3_Al, as in a suspension, which leads to cross‐linking, formation of insoluble aluminoxane as reported in the case of Et_3_Al,^[5]^ and ultimately formation of a swollen, boehmite or alumina gel^[6]^ if sufficient water is present.

The hydrolysis of Me_3_Al has been studied in detail, both experimentally and also theoretically. At low temperatures and in a donor solvent (i. e. a solvent that can act as a neutral Lewis base towards Me_3_Al), which moderates the hydrolysis reaction, the principle products formed are (Me_2_AlOAlMe_2_)_n_ or (Me_2_AlOH)_n_ (n≥2) depending on stoichiometry.^[7]^ (Me_2_AlOAlMe_2_)_n_ has never been obtained in pure form, is predominantly trimeric in solution, and is known to form h‐MAO+Me_3_Al when distillation is attempted.^[8]^ (Me_2_AlOH)_n_ is unstable at room temperature in donor solvents and is said to form MAO but with unusual characteristics such as 5‐coordinate Al, with residual OH groups.^[9]^ While this material is not useful as an activator, it does serve as a precursor to aluminoxane carboxylates and related materials.[^6^, ^9^] It is known in the case of Et_3_Al that when the initial hydrolysis in non‐donor media is controlled, insoluble aluminoxane of this type can be converted to soluble aluminoxane through addition of an equimolar excess of Et_3_Al and also contains residual OH groups.^[5]^

In non‐donor solvents, the preparation and composition of MAO has been studied at low temperature using both solvent fractionation, cryoscopy and NMR by the group of Sinn and co‐workers.^[10]^ They were able to demonstrate that low temperature hydrolysis, involving an excess of Me_3_Al, furnished low MW oligomeric material, as might be expected for a classical, step‐growth condensation. Sinn invoked aggregation of these materials via dative Al−O interactions to form higher MW MAO as was hypothesized earlier.^[7a]^ Based on the reports of Barron and co‐workers on the structure of t‐butylaluminoxanes,^[11]^ Sinn and co‐workers invoked a cage structure formed via aggregation of a linear tetramer (i. e. Me_2_Al(OAlMe)_3_OAlMe_2_) for the active component of MAO. We should note that Sinn and co‐workers adopted a different nomenclature based on the number of Al atoms (i. e. a Sinn pentamer) which we will use going forward.

Pioneering theoretical work by Zurek and Ziegler examined cage structures for h‐MAO^[12]^ and also studied the reaction of these cages with Me_3_Al,^[12a]^ motivated by the work Barron and co‐workers, who demonstrated that Me_3_Al was required for effective catalyst activation using strained, *t*‐butylaluminoxane cages.^[13]^ Other work has shown that models such as nanotubes^[14]^ have comparable stability to classical cages with formula (MeAlO)_n_.^[15]^ In order for cages to be as stable (per mole repeat unit) they must be of large size so that strained Al_2_O_2_ rings are minimized.[^12^, ^13^, ^14^, ^15^, ^16^] Generally speaking, such stable structures reversibly bind only small amounts of Me_3_Al.[^12a^, ^14^]

**Scheme 1:**
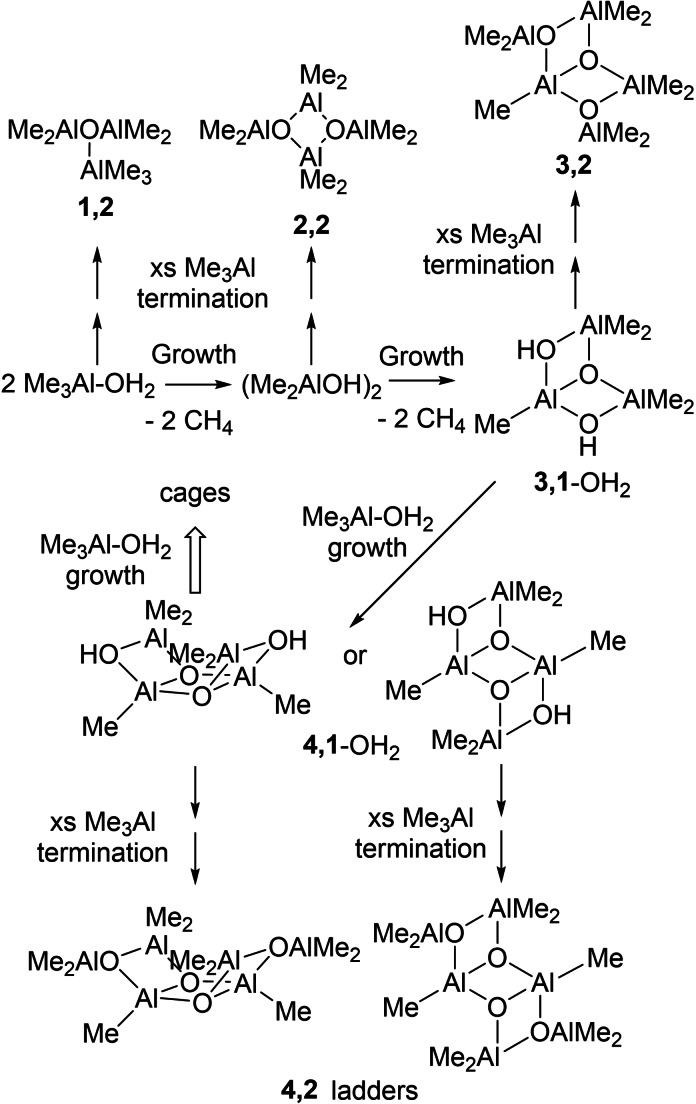
Hydrolysis of Me_3_Al as studied by Hall and co‐workers.^[17]^

Other theoretical work has examined the step‐wise hydrolysis of Me_3_Al at various levels of theory. One of the few papers to look at both the thermodynamics and dynamics of this process was reported quite some time ago by Hall and co‐workers (Scheme 1).^[17]^ Glaser and Sun looked at the thermodynamics of various initial steps, motivated by the early experiments of Sinn and other workers, and reached different conclusions about the mechanism of growth in the presence of donors.^[18]^ Linnolahti and co‐workers have systematically studied the growth reaction,^[19]^ finally reaching the size of large cages in the size domain of h‐MAO (typically 1000–3000 g mol^−1^).^[20]^

MAO prepared by hydrolysis reacts with chelating donors such as OMTS to form ion‐pairs [Me_2_Al(OMTS)]^+^ [(MeAlO)_n_(Me_3_Al)_m_Me]^−^ which can be detected and characterized by ESI‐MS.^[21]^ In recent papers, we reported ESI‐MS experiments monitoring the hydrolysis of Me_3_Al in fluoroarene solvents.^[22]^ Oligomeric, aluminoxane‐based ion‐pairs (n=6–10, m=4) rapidly appeared within minutes upon mixing of Me_3_Al with water and the composition of the mixture evolved on a longer time scale (tens of minutes to hours) to furnish a simpler mixture of ionic species. The final anion distribution was dominated by an anion with *m/z* 1375 with composition [(MeAlO)_16_(Me_3_Al)_6_Me]^−^ – hereinafter [**16,6**]^−^.

In subsequent work it was shown that the change in composition of these mixtures had at least two underlying rates, the slowest of which seemed related to aging of MAO based on the longer term changes in ion speciation seen in these mixtures,^[23]^ compared with early studies on aging of commercial MAO.^[21b]^ In addition, h‐MAO or non‐hydrolytic MAO prepared using different synthetic methods, under controlled conditions was shown to have different anion distributions, thus illustrating that the composition of the hydrolysis mixtures upon completion reflected the specific conditions of those reactions.

Most recently, we showed that there was very good agreement between ion intensities of these final mixtures as measured by ESI‐MS and what would be predicted based on both the stability and reactivity of likely neutral precursors, undergoing ionization by the most favourable process.^[24]^ In general, for high MW anions like [**16,6**]^−^ the preferred pathway is [Me]^−^ abstraction from Me_3_Al‐OMTS, while the most stable anions are derived from less stable, lower MW sheets such as **7,5** by the process of [Me_2_Al]^+^ abstraction. In practice it is difficult to distinguish between these ionization processes^[19]^ at least by experiment.

These theoretical and experimental results, especially those involving reaction of h‐MAO with R_3_Al,^[25]^ implicate sheets, containing 5‐ or 4‐coordinate Al, with structural Me_3_Al^[26]^ incorporated along their edges, as the likely structures for the reactive components of h‐MAO. Furthermore, some of the transient behavior seen during hydrolysis of Me_3_Al is interpretable, based on anion vs. neutral stability.^[24]^ However, important questions remain as to the likely processes involved in these growth reactions. In this paper, we delineate some features of the growth reactions studied earlier through both theory and modeling.

## Results and Discussion

Before we present our results, we would like to briefly review the work of Hall and co‐workers^[17]^ since that paper (hereinafter NHBS) is important to the discussion of the current results. In this work, NHBS postulated that formation of oligomers occurred by competing processes involving monomeric Me_3_Al‐OH_2_ formed reversibly from Me_6_Al_2_. The growth steps involve reaction of Me_3_Al‐OH_2_ with itself, Me_3_Al, or higher MW oligomers to form species with μ‐OH groups as summarized in Scheme 1.^[17]^

All of these growth steps feature fairly low activation energies (*E_a_* =9.2–50 kJ mol^−1^) consistent with experimental results. On the other hand, termination steps involve reaction of these same intermediates with Me_3_Al, and those steps possess even lower barriers (*E_a_* =2.1–3.5 kJ mol^−1^) dominated by the entropy loss in the transition state. Finally, two morphologies were identified for low MW oligomers; open ladder structures vs. “nido” cage precursors, ultimately leading to small and then larger cages. Though cage precursors were favoured thermodynamically over open, ladder structures by 9.2 kJ mol^−1^, the initial growth reactions leading to the cage precursors featured significantly higher activation energies (*E_a_* =58.5 vs. 25.1 kJ mol^−1^ for the rate determining steps). On this basis one would predict a kinetic preference for ladders of about 700,000 : 1 at 298 K as termination is much faster than growth, especially in the presence of a local excess of Me_3_Al.

## Initial Hydrolysis Steps

The original sequence of reactions studied by NHBS involved the following basic steps at the very start (Eq. 1):

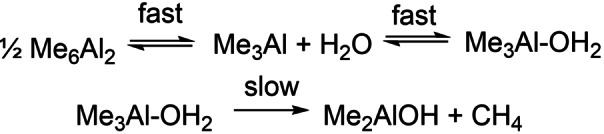




The first dissociation equilibrium has been studied in detail both experimentally^[27]^ and theoretically^[28]^ but as far as we are aware only NHBS looked at the dynamics. The process was characterized by very high barriers with activation energies of 61.5 (dimerization) and 133.5 (dissociation) kJ mol^−1^ at the MP2/6‐31G** level of theory. The difference in these E_a_ values (72.0 kJ mol^−1^ ∼ ▵*H*_o_) is in reasonable agreement with the experimental values for dissociation in gas phase (▵*H*_o_=85.4 kJ mol^−1^ with ▵*S*_o_=180.3 J mol^−1^ K^−1^).^[29]^

However, it is known from experiment that ▵*H* ^≠^ for exchange of terminal and bridging methyl groups in Me_2_Al_6_ is about 63–71 kJ mol^−1^ in hydrocarbon or toluene solution at −50 °C. A mechanism involving dissociation into monomeric Me_3_Al is widely accepted for this exchange process, though the solvent may play a role as the energetics differ significantly between hydrocarbon and toluene solution. Reanalysis of the original line shape data^[30]^ in cyclopentane using the Eyring relationship gives ▵*H* ^≠^=72.8 kJ mol^−1^ with ▵*S* ^≠^=107 J mol^−1^ K^−1^ for the rate‐determining, dissociation step. Since the temperature range of these experiments was only 10 °C, the entropy value is a rough estimate. However, on this basis one would expect ▵*G* ^≠^=40.7 kJ mol^−1^ at 298 K in solution.

Also, from the temperature dependence of the dissociation equilibrium in hydrocarbon solution, giving ▵*G*=32.0 kJ mol^−1^ at 298 K, the free energy barrier to dimerization in solution (∼8.7 kJ mol^−1^) appears dominated by the loss of entropy in the transition state. From a practical perspective, it is widely appreciated that dimerization of Me_3_Al occurs at essentially diffusion controlled rates in solution at all temperatures (*k*_d_∼7.56×10^9^ M^−1^ s^−1^ at 298 K).[^27^, ^31^]

We examined the dissociation of Me_6_Al_2_ at the M06‐2X^[32]^/TZVP^[33]^ method as implemented in Gaussian 16^[34]^ and though we could not locate a stable transition structure at this level of theory, the enthalpy and entropy changes associated with increasing the Al−Al distance show an inflection point that leads to a maximum in free energy at roughly 40.4 kJ mol^−1^ in excellent agreement with the extrapolated value of 40.7 kJ mol^−1^ based on the experimental data in cyclopentane solution (Supporting Information, Tables S1 and S2).

The high barriers for Me_6_Al_2_ dissociation led NHBS to conclude that formation of Me_3_Al‐OH_2_ via reaction of H_2_O with monomeric Me_3_Al was not competitive with a bimolecular process involving Me_6_Al_2_ leading to this same species. We also re‐examined this process and find that the transformation of Me_6_Al_2_+H_2_O to the final product Me_5_Al_2_(μ‐OH)+CH_4_ occurs with rate‐determining formation of a high energy intermediate Me_6_Al_2_ ⋅ OH_2_ with ▵*G* ^≠^=64.5 and *E_a_* ≈Δ*H* ^≠^+*RT*=14.8 kJ mol^−1^ in gas‐phase, considerably lower than their estimate of *E_a_* =26.8 kJ mol^−1^ based on a two‐step process involving Me_3_Al‐OH_2_. Further, they did not consider unimolecular decomposition of Me_3_Al‐OH_2_ to form Me_2_AlOH+CH_4_ as kinetically relevant, despite that it too has a fairly low ▵*G* ^≠^=60.2 kJ mol^−1^ in gas phase at 298 K.

The consequences of these differences can be rigorously examined through numerical simulation of the various competing processes using the COPASI software,[^35^, ^36^] subject to certain simplifying assumptions. The first of these is that any process involving monomeric Me_2_AlX (X=Me or OH) forming a dimeric species or a tetrahedral intermediate (e. g. Me_3_Al‐OH_2_) is essentially diffusion controlled, but possibly reversible, while methane elimination from various species is chemically controlled but irreversible. Using these assumptions and our estimates for the free energy barriers at 298 K, one can show (Supporting Information Figure S1) that the only kinetically relevant steps for the initial hydrolysis of Me_3_Al are those shown in Equation (1).

In other words, Me_3_Al‐OH_2_ is not a plausible monomer for formation of ladder or cage structures, though one might argue, by analogy, that Me_2_AlOH is (see however below). Importantly, decomposition of Me_3_Al‐OH_2_ is rate‐determining compared to all other initial steps. Conversely, unimolecular decomposition of species like Me_5_Al_2_(μ‐OH), Me_2_Al(μ‐OH)_2_AlMe_2_ or higher order species of this type feature significant barriers to methane elimination (▵*G* ^≠^>100 kJ mol^−1^ Supporting Information Table S3) and to the point where bimolecular reaction involving Me_3_Al (or even Me_6_Al_2_) with these intermediates, as invoked by NHBS, appears entirely relevant as a mechanism for formation of neutral aluminoxanes. These so‐called termination events, to use the terminology adopted by NHBS, do feature very low barriers (▵*G* ^≠^=31.7 kJ mol^−1^ for the reaction of Me_5_Al_2_(μ‐OH)+Me_3_Al at the M06‐2X/TZVP level of theory).

## Simulation of the initial hydrolysis steps

**Scheme 2:**
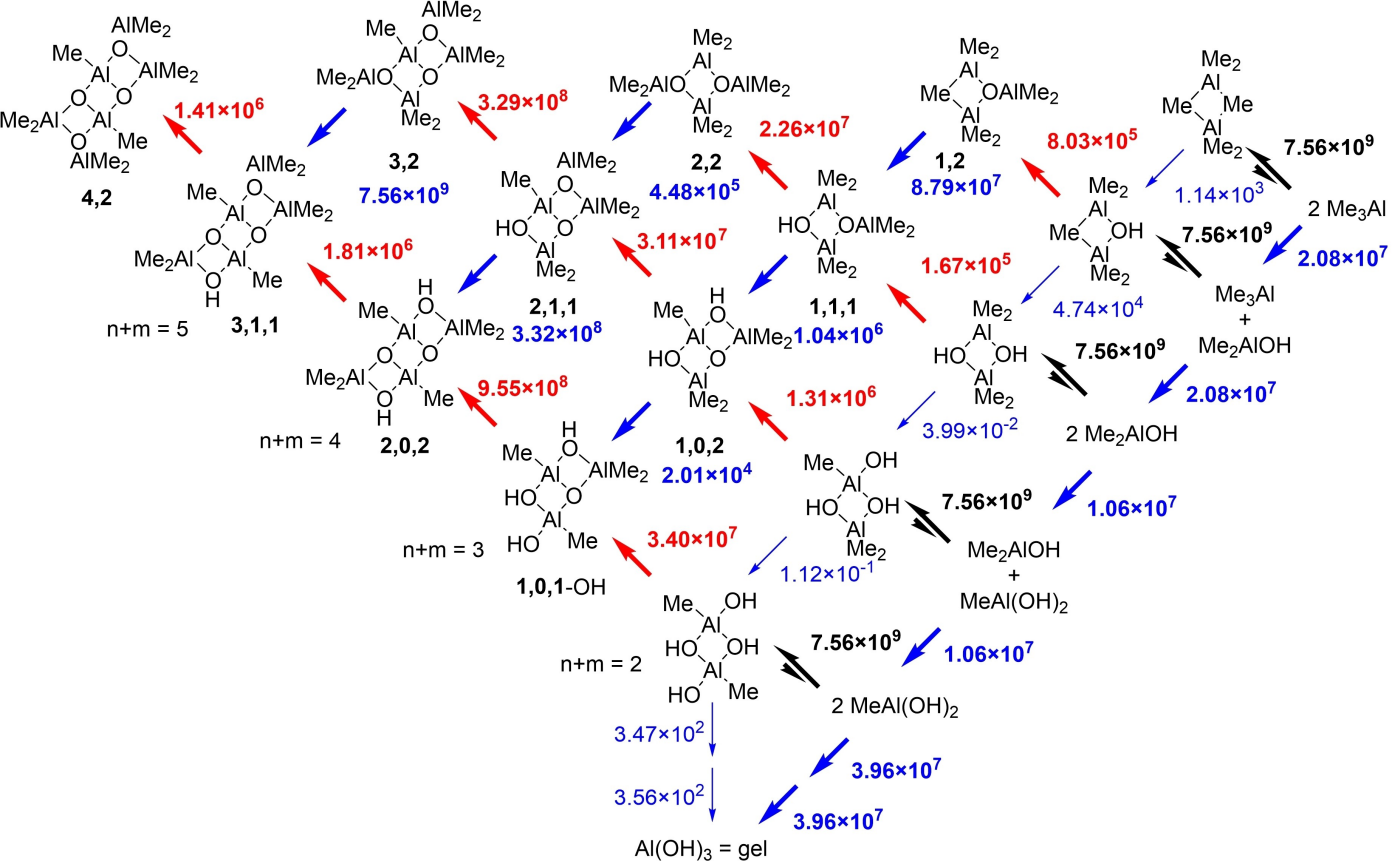
Network of Competing Hydrolysis and CH_4_ elimination steps.^[16]^ The ladder structures are numbered according to the convention (MeAlO)_n_(Me_3_Al)_m_ for aluminoxanes and (MeAlO)_n_(Me_3_Al)_m_(Me_2_AlOH)_o_ for intermediates involved in their formation. Rate constants used in the simulation are in M^−1^ s^−1^.

The hydrolysis of Me_3_Al involves a network of reactions involving competing elementary steps. These steps, the corresponding intermediates and final products have been documented for n≤4^[16]^ and are depicted in Scheme 2 where blue arrows represent hydrolysis (of both trigonal and tetrahedral AlMe), and the red arrows involve reaction of OH intermediates with Me_3_Al. Bold arrows indicate rapid steps occurring at competitive rates, while much slower steps are in plain.

It should be mentioned that the final products of this network, specifically **4,2** and **3,2** correspond to the ladder structures studied by NHBS. However, these are not the most stable structures for *n*=4 and 3,^[20]^ nor even the most stable isomer of **4,2** identified by theory.^[16]^ Our intention here is to illustrate the complexity as well as features of this initial network of reaction using the frame of reference established by NHBS.

The simple two‐step reaction for hydrolysis of Me_3_Al, leading to Me_2_AlOH will be governed by the rate determining decomposition of Me_3_Al‐OH_2_. Binding of water to Me_3_Al is reversible but favorable with ▵*G*=−29.0 kJ mol^−1^ and K=1.19×10^5^ M^−1^ at 298 K. One expects no barrier to binding and so the forward reaction will occur at the rate of diffusion with constant k_1_=7.56×10^9^ M^−1^ s^−1^ giving k_‐1_≤6.3×10^4^ s^−1^ This is obviously faster than unimolecular decomposition forming Me_2_AlOH+CH_4_ (k_2_=173 s^−1^ at 298 K). If we apply the steady state approximation to the intermediate, the overall rate constant for this process is given by k_1_k_2_/(k_‐1_+k_2_)=2.08×10^7^ M^−1^ s^−1^. We will use simplifications of this sort going forward.

The initial product Me_2_AlOH which is unstable as a monomer, and will suffer three competitive reactions that occur at more or less diffusion controlled rates, binding of Me_3_Al (to form Me_5_Al_2_(μ‐OH)) dimerization (to form Me_2_Al(μ‐OH)_2_AlMe_2_), and reversible binding of water, followed by slower irreversible methane elimination. The first two processes are strongly exergonic with ▵*G*=−91.2 and −186.1 kJ mol^−1^ and can be considered practically irreversible. As before binding of water is exergonic (▵*G*=−39.3 kJ mol^−1^) and the overall rate of binding and methane elimination feature *k*=1.06×10^7^ M^−1^ s^−1^ and leads to MeAl(OH)_2_+CH_4_. A final hydrolysis step to form Al(OH)_3_ (i. e. gel) is significantly less exergonic than the first two, while binding of water to MeAl(OH)_2_ can be considered irreversible with ▵*G*=−51.5 kJ mol^−1^. The last hydrolysis step (of monomeric Me_x_Al(OH)_3‐x_) is the most rapid of the three with k=3.96×10^7^ M^−1^ s^−1^. As before, MeAl(OH)_2_ can presumably bind to itself, or to other monomeric R_3_Al present and each of these steps is essentially irreversible and diffusion controlled.

Di‐ and poly‐nuclear intermediates featuring μ‐OH groups are expected to be rather stable with respect to methane elimination, at least in a unimolecular sense, with barriers in excess of 100 kJ mol^−1^. It is less clear that species with terminal OH groups will be as stable but for the sake of argument we assume they are in what follows. Shown in Scheme 2 are the various possible dinuclear species (*n+m*=2). By analogy to Me_6_Al_2_ (see above) they can form sequentially from Me_5_Al_2_OH via much slower hydrolysis (thin blue arrows), leading ultimately to 2 equiv. of Al(OH)_3_. Alternately, faster reaction with Me_3_Al (red arrows) affords ultimately soluble aluminoxanes.

The elementary steps shown in the Scheme, along with the direct hydrolysis of Me_3‐x_Al(OH)_x_ (x=0–2) were simulated using the COPASI numerical simulator with the simplifying assumptions that dimerization events were diffusion controlled and irreversible (except in the case of Me_6_Al_2_) while binding of water to trigonal Al, also proceeded at diffusion controlled rates and was considered reversible, based on the magnitude of ▵*G* (see above). Methane eliminations were treated as single steps using the steady state approximation as outlined above and with the rate determining step involving the second in most cases.

**Figure 1:**
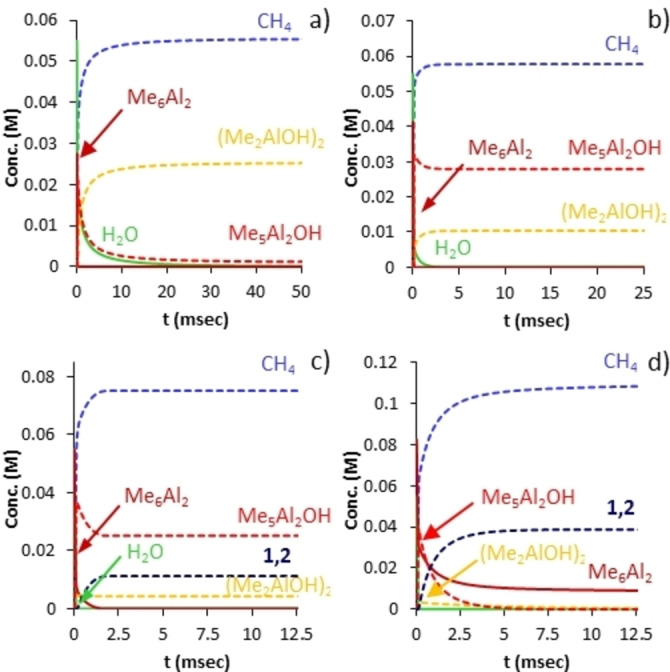
Consumption of starting materials (solid lines) and product formation (dashed lines) vs. time for the solution hydrolysis ([H_2_O]=0.055 M) of Me_3_Al. a) Al : O=1 : 1 b) Al : O=1.5 : 1 c) Al : O=2 : 1 d) Al : O=3 : 1.

Shown in Figure 1 are the simulation results for various species of interest and with [H_2_O]=[Me_3_Al]=0.055 M (i. e. [Me_6_Al_2_]=0.0275 M). Consumption of free water (green curve) is complete within 100 msec ‐ assuming homogeneous conditions, and efficient mixing. Consumption of Me_6_Al_2_ (dark red) occurs much faster; evidently, none of the monitoring experiments[^22^, ^23^] were detecting initial hydrolysis of Me_3_Al as this process is complete on the time scale of milliseconds.

The major product formed under these conditions is not soluble aluminoxane but instead (Me_2_AlOH)_2_ (or higher oligomers orange dashed curve) which forms both initially and also at the expense of Me_5_Al_2_OH (red dashed curve) via direct hydrolysis. Small amounts of aluminoxanes containing OH groups are present (not shown in Figure 1a), total concentration ca. 0.45 mM), with the major products being **2,0,2** and **1,0,1**‐OH (Scheme 2). The only aluminoxane present in *detectable* amounts is ladder‐**4,2** (ca. 1.7×10^−7^ M). Needless to say a 1 : 1 stoichiometry does not furnish soluble MAO in any significant quantities.

These conclusions are unaffected by the Al : O ratio within limits. For example, with a 50 mol% excess of Me_3_Al ([Me_6_Al_2_]=0.04125 M), comparable amounts of (Me_2_AlOH)_2_ and Me_5_Al_2_OH are produced, though the latter predominates. Even at a 2 : 1 stoichiometry (Me_2_AlOH)_2_ and intermediates containing OH groups are still present, though soluble aluminoxanes, principally **1,2** (i. e. Me_2_AlOAlMe_2_, complexed by Me_3_Al) are now dominant in the mixture. At this stoichiometry, there is a continuous excess of Me_6_Al_2_ over water, whereas the converse is true at lower Al : O ratios. At a 3 : 1 stoichiometry, most of the OH‐containing intermediates are consumed, though some residual (Me_2_AlOH)_2_ remains, while a mixture of **1,2**, with **2,2** in lower amounts is formed. This feature is preserved at higher amounts of Me_3_Al, though little (Me_2_AlOH)_2_ would be formed at a 4 : 1 ratio.

It is generally known that compounds such as (Me_2_AlOH)_n_ or Me_5_Al_2_OH are unstable at room temperature, and yet the simulation predicts that these compounds would dominate in the mixture at short time scales. With barriers to unimolecular CH_4_ elimination of at least 100 kJ mol^−1^, these compounds would have $1\;/\;\;2$ ‐lives of nearly 10 h at 298 K and might even be isolable.

This is certainly the case for direct hydrolysis of ^*t*^Bu_3_Al, where both [^*t*^Bu_2_AlOH]_3_ (**I**) and [^*t*^Bu_2_AlOAl^*t*^Bu_2_]_2_ (**II** structure analogous of **2,2** in Scheme 2) have been isolated and structurally characterized.^[6]^ Thermolysis of pure [^*t*^Bu_2_AlOH]_3_ affords strained aluminoxane cages derived by the bimolecular condensation of [^*t*^Bu_2_AlOH]_3_ to form a mixture of *inter alia* (^*t*^BuAlO)_n_ with n=6, 9 and 12. On the other hand, hydrolysis of ^*t*^Bu_3_Al using a hydrated salt followed by thermolysis affords a mixture of compounds of which **II** and (^t^BuAlO)_8_ (**III**) predominate suggesting a different fate when **I** (or ^*t*^Bu_2_AlOH) is generated in the presence of ^t^Bu_3_Al. On the theoretical front species such as (Me_2_AlOH)_n_ (n=3 or 4) are also considered plausible precursors for formation of much more stable nanotubes,^[14]^ though some of the barriers for that process are also predicted to be substantial.^[37]^

We examined the binding of (Me_2_AlOH)_2_ or Me_5_Al_2_OH with Me_6_Al_2_ thinking their direct association might be competitive with steps involving monomeric Me_3_Al, for which no barrier is predicted but the equilibrium concentration of monomer is very low relative to Me_6_Al_2_. These reactions are facile with barriers in the range 50–60 kJ mol^−1^ for the dinuclear Al‐OH species (*n*+*m*=2) shown in Scheme 2. It should be mentioned that this alternate pathway is also available to any other intermediate bearing an OH group. We did incorporate this into the model but it did not alter the fundamental results. That is principally because Me_6_Al_2_ is used up rapidly under stoichiometric condition, while both (Me_2_AlOH)_n_ or Me_5_Al_2_OH are much more reactive towards monomeric Me_3_Al when excess Me_6_Al_2_ is present.

**Scheme 3:**
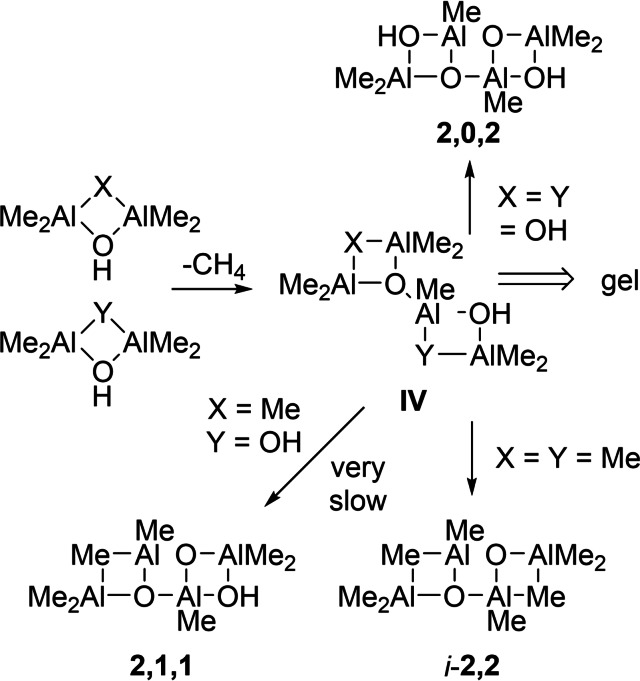
Bimolecular condensation of (Me_2_AlOH)_2_ and Me_5_Al_2_OH.

We have examined other possible reaction pathways of (Me_2_AlOH)_2_ or Me_5_Al_2_OH. For example, either might react with excess Me_6_Al_2_ to furnish in the case of Me_5_Al_2_OH, either **1,2**+Me_3_Al (or more stable **1,3** see below) and methane. We calculated barriers of 110.8 and 113.3 kJ mol^−1^ for this initial reaction, involving Me_5_Al_2_OH and (Me_2_AlOH)_2_, respectively. The only process studied that is even roughly competitive is the reaction of (Me_2_AlOH)_2_ with itself, producing the species **IV** shown in Scheme 3 (X=Y=OH) with a barrier of 78.1 kJ mol^−1^. This species can undergo intramolecular CH_4_ elimination to form **2,0,2** but with a more significant barrier of 87.1 kJ mol^−1^.

Note that **2,0,2** can exist in ladder or nido form and the nido structure (2 equiv.) might form (MeAlO)_8_ with a structure analogous to that proposed for (^t^BuAlO)_8_.^[6]^ Though the transition state forming the ladder form (87.1 kJ mol^−1^) is significantly lower in energy than that for forming the nido form (101.9 kJ mol^−1^) it is possible for the ladder form to isomerize to the more stable nido form (ΔΔ*G*=−9.3 kJ mol^−1^) via ring opening and this barrier is surprisingly low (64.1 kJ mol^−1^). Thus, cage precursors can still be generated in this manner. We note that formation of a **4,0** cube from the nido **2,0,2** structure is kinetically quite unfavourable (Δ*G* ^≠^=143 kJ mol^−1^) so absent reaction with Me_3_Al, further hydrolysis, or self‐condensation this compound would persist in solution.

Reaction of (Me_2_AlOH)_2_ with Me_5_Al_2_OH, or condensation of Me_5_Al_2_OH with itself are also feasible. In these two cases, the products are expected to form **2,1,1** and *i*‐**2,2** via subsequent intramolecular elimination of CH_4_ (Scheme 3). Formation of **2,1,1** involves two CH_4_ elimination steps with barriers of 87.1 and 88.3 kJ mol^−1^ if (Me_2_AlOH)_2_ acts as a proton source (X=OH, Y=Me), while the other scenario (X=Me, Y=OH), involves barriers of 72.5 and 102.9 kJ mol^−1^. Since the first step for the latter process is much lower in energy than the alternative, while the second step is over 100 kJ mol^−1^ it is clear that the intermediate formed (**IV** X=Me, Y=OH *i*‐**1,1,2**) would dominate and persist in solution. Formation of *i‐* **2,2** from Me_5_Al_2_OH involves barriers of 80.5 and 86.2 kJ mol^−1^ while this aluminoxane is 41.1 kJ mol^−1^ higher in energy than **2,2** and would isomerize readily.

**Figure 2:**
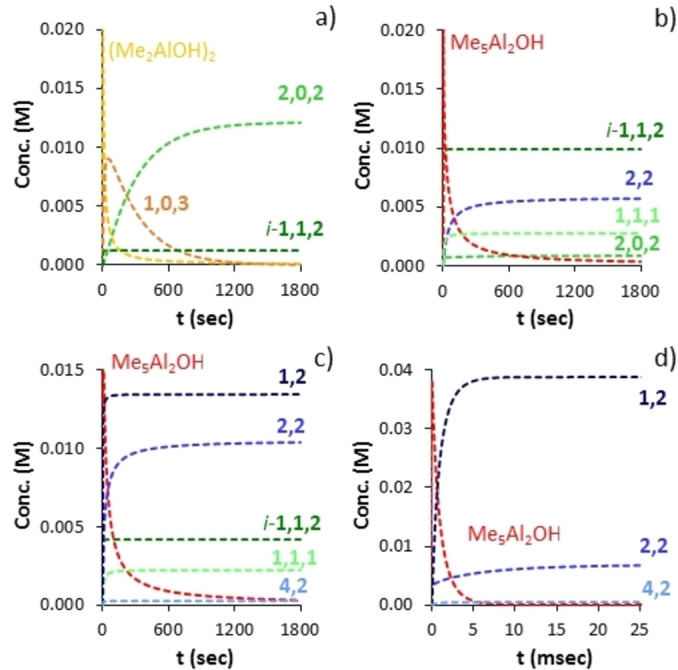
Production of aluminoxanes and other products vs. time, including bimolecular condensations of (Me_2_AlOH)_2_ and Me_5_Al_2_OH (Scheme 3). For conditions and stoichiometry see Figure 1.

We incorporated these reactions into the model and the results are depicted in Figure 2. Under stoichiometric conditions and after 30 min at 298 K, the main product is **2,0,2** formed almost exclusively via the reaction in Scheme 3. At 50 mol% excess of Me_3_Al the principal kinetic product is now Me_5_Al_2_OH and so **2,2** is formed instead by this slower process. The *i*‐**1,1,2** species persists as one ramps up the amount of Me_3_Al as it is formed most rapidly from Me_5_Al_2_OH and (Me_2_AlOH)_2_. At a 3 : 1 stoichiometry we return to the same conditions as in Figure 1; very rapid formation of mainly **1,2** with lesser amounts of **2,2** and very little **4,2** (or **3,2**).

Note that structure **IV** (X=Y=OH) is an A_3_B_3_ type of monomer^[38]^ assuming OH and AlMe_2_ groups are the reactive groups present with respect to further methane elimination. The usual result of a step‐growth reaction involving an A_3_B_3_ monomer is an insoluble, network polymer. This may be related to the formation of insoluble, aluminoxane gels that are always generated during hydrolyses of either Me_3_Al (or Et_3_Al) under near stoichiometric conditions. We suspect the other multi‐functional intermediates such as **2,0,2** or i‐**1,1,2** are also capable of gelation, either on their own, or certainly through reaction with other multi‐functional intermediates. Conversely, Me_3_Al‐rich species such as **1,2** (or it's more stable congener **1,3**) might serve as a source of Me_3_Al leading to aluminoxane gel depleted in OH groups.

Not much is known about aluminoxane gel other than it does contain residual OH groups and can be dissolved upon addition of sufficient Me_3_Al (usually a full equivalent with respect to water initially present). In our monitoring studies we never detected anionic species with OH groups, though these form during adventitious hydrolysis of commercial MAO solutions so we can't exclude their formation. Certainly, OH groups are vanishingly small in commercial MAO by other spectroscopic methods.

In essence, to produce high yields of soluble aluminoxane, one must be working with a significant excess of Me_3_Al over dissolved water, and these conditions (e. g. slow or controlled addition of water to Me_3_Al) reflect how MAO is prepared both in the laboratory and under industrial conditions. The initial product distribution is characterized by low MW oligomers – see Figure 2c)‐d).

## Aggregation Mechanisms

**Figure 3:**
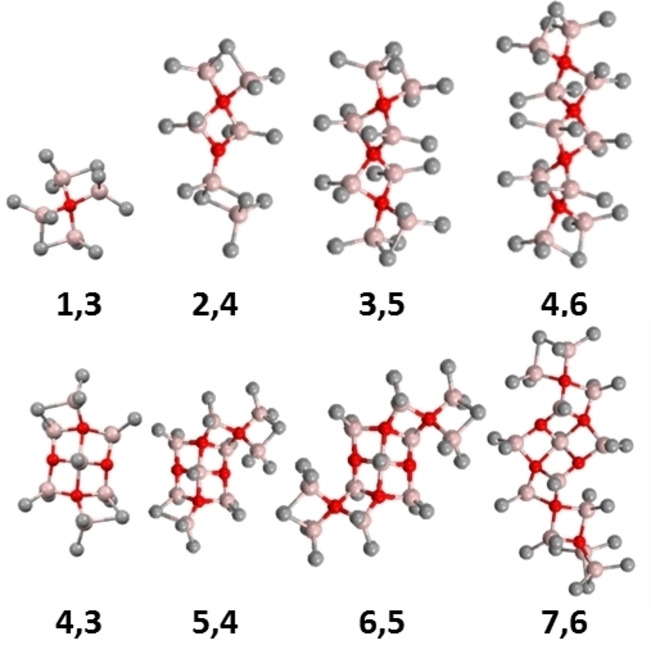
Top: Most stable aluminoxane chains identified by theory. Bottom: Most stable sheets for n=4‐7.

While formation of large sheets like neutral **16,6** is certainly driven by thermodynamics[^23^, ^24^] one may ask how these large sheets form? If we look more closely at the most stable structures in the size range n=1–4 vs. 4–7 (Figure 3)^[20]^ one can see that the chains are really just aggregates of the Sinn dimer (Me_2_AlOAlMe_2_) end‐capped with Me_3_Al.

In fact, in the size range n=1–4, aggregates of **1,1** are involved in stable structures such as **3,3** and **4,4** which are the cyclic trimer and tetramer of **1,1**. While they are important as a source of **1,m** (m=2,3) or even Me_3_Al, they cannot form 2 dimensional structures like sheets without rearrangement. In contrast, the stable 5‐coordinate sheets are built up of both the Sinn dimer and the Sinn trimer, stabilized by binding of Me_3_Al. The latter structures are likely precursors to the lowest MW anions detected experimentally.^[24]^

Various unsaturated aluminoxanes would be in equilibrium with **1,3** and **2,4** through reversible binding of Me_3_Al. Free energy differences suggest that small amounts of **1,2** (and $1\;/\;\;2$ Me_6_Al_2_) will be in equilibrium with **1,3** (*K*_eq_=3.9×10^−3^ M), and that the same is true for **2,2** and **2,3** with respect to **2,4** (*K*_eq_=1.1 and 9.6×10^−3^ M, for dissociation of **2,3** and **2,4** respectively). Thus, aggregation of these species to form sheets is one possible mechanism for forming the precursors whose anions are detected by experiment.

As delineated in the Supporting Information (pgs. 5–8), monitoring of the hydrolysis of Me_3_Al leads to rapid appearance of anions with n=6–10 and m=4 upon quenching with OMTS (Supporting Information, Figures S3 to S5). These anions appear with variable relative intensity depending on solvent, and importantly the Me_3_Al:H_2_O ratio used in these experiments. These anions then disappear with formation of higher MW anions, the more prominent of which have the compositions n=14‐18 with m=5 or 6. At longer time scales, particularly noticeable in *o*‐DFB, conversion of odd‐numbered anions n=15 and 17 to even numbered anions 16 and 18 occurs at a distinctly slower rate. The final mixtures are dominated by even‐numbered anions (Supporting Information, Figure S6). In earlier work, aging of MAO also involves conversion of even‐numbered anions to even‐numbered anions, at least in the initial stages.^[21b]^

This result (a preponderance of even numbered ions upon completion) is inconsistent with a growth mechanism involving step‐wise hydrolysis of Me_3_Al. That process has been studied in depth by computation, and there is no indication that even‐numbered sheets or cages are favoured thermodynamically over odd‐numbered structures. In our opinion this strongly speaks to the mechanism of sheet formation.

**Scheme 4:**
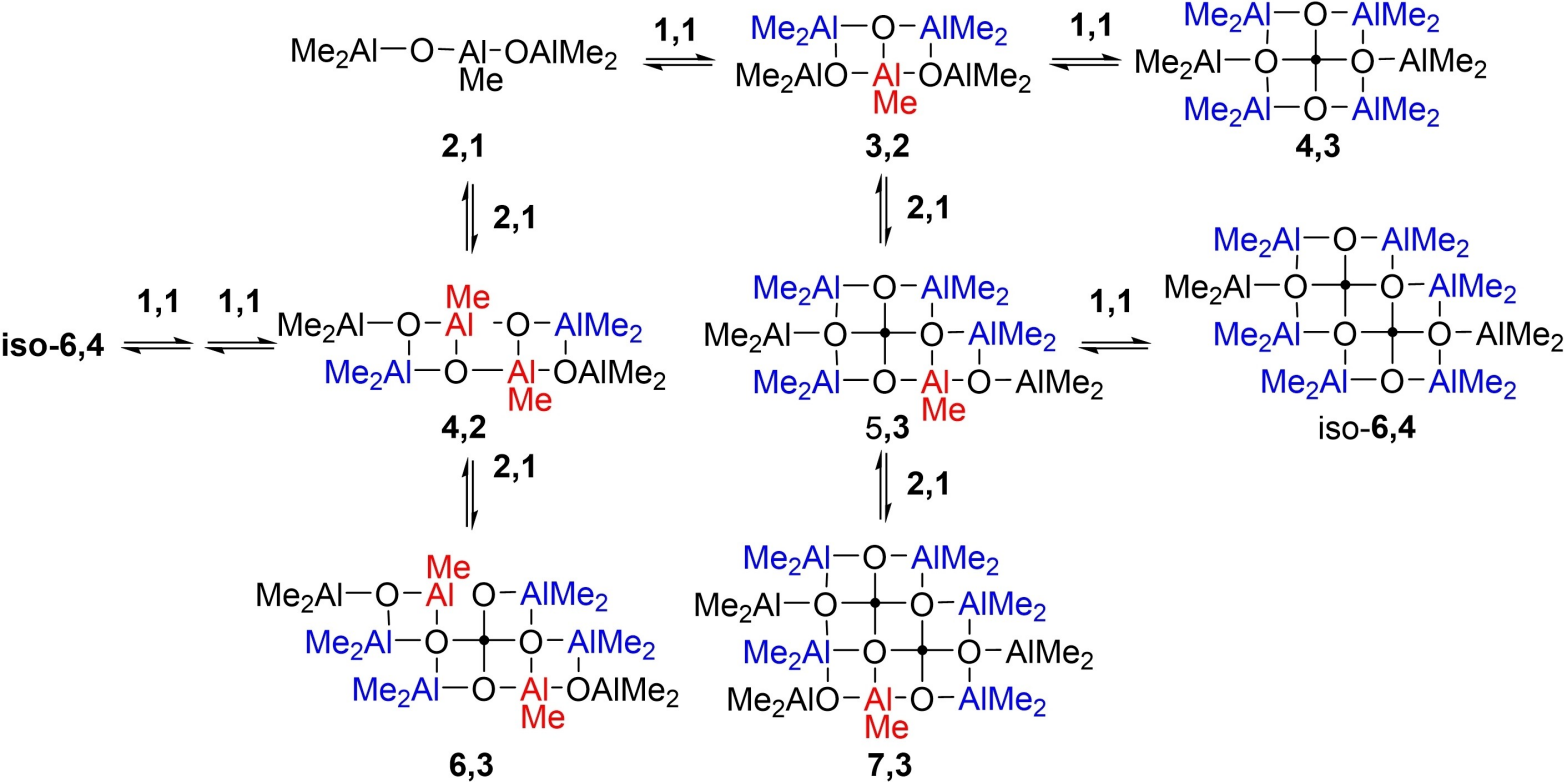
Aggregation of linear aluminoxane oligomers into sheets.

Consider a situation where the principle products of controlled hydrolysis are the simple Sinn dimer (Me_2_AlOAlMe_2_) and trimer Me_2_Al(OAlMe)_2_Me. This is the expected outcome of a controlled hydrolysis at low temperature and using an excess of Me_3_Al (e. g. Figure 2d). If we consider possible aggregation steps involving these materials (Scheme 4), it becomes obvious that only the Sinn trimer leads to structures which can continue to grow by additional binding of Sinn dimer or trimer along *both* edges (with red AlMe groups) to form sheets with 5‐coordinate Al.

Alternately, growth along an edge of the sheet is curtailed when the Sinn dimer reacts at that site (leading to blue coloured, tetrahedral O_2_AlMe_2_ groups). However, two such steps are required for sheets that are even in number vs. only one step for an odd number. In any event, as sheets comprised of 5‐C Al at the centre and 4‐C Al and 3‐C or 4‐C O at the edges are stable end products identified by theory, it is obvious the termination of their growth by this mechanism will produce predominantly even numbered sheets.

Also, if one considers fusion of two even numbered sheets, each with two reactive edges, the product of such a reaction will also have two reactive edges. In contrast, reaction of an odd‐numbered sheet with an even numbered sheet gives rise to an odd‐numbered sheet with only one reactive edge, while the combination of two odd sheets gives an even numbered sheet which cannot readily grow further by the processes illustrated. The prediction is again a preference for even‐numbered products.

These simple concepts explain the unusual feature of the ESI‐MS results. The problem is that species like **1,1** or **2,1** are far too unstable to be viable intermediates. The concentration of Sinn dimer in an equilibrium mixture of **1,3** and **1,2** would be 3.9×10^−17^ M for [Al]=0.055 M (**1,1**+Me_6_Al_2_ is 97.5 kJ mol^−1^ less than stable than **1,3**). Any reaction involving **1,1** corresponds to aggregation with an initial half‐life of about 4 weeks at these concentrations and at diffusion‐controlled rates!

**Figure 4:**
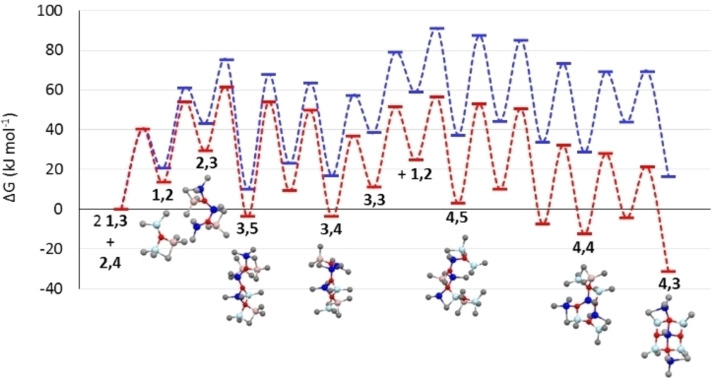
Energetics of formation of sheet **4,3** by aggregation of linear oligomers **1,3** (2 equiv.) and **2,4**. Total free energies are referenced with respect to starting materials and Me_6_Al_2_ (red curve) or Me_3_Al (blue curve) as the by‐product. For details of the pathway see Supporting Information Table S4 and Figure S7.

We invoke similar assembly processes here but involving, as stable intermediates, structures related to those found computationally. For example, aggregation of **1,2** (in equilibrium with **1,3** through loss of Me_3_Al) with **2,3** (also accessible from **2,4** with some rearrangement) could form chains **3,5**, **3,4** and ultimately **3,3** through loss of Me_3_Al (Figure 4).

With the exception of one rearrangement for which the activation free energy is 57.8 kJ mol^−1^ involving isomerization of **3,5** to a higher energy isomer, the other steps involve dissociation of Me_3_Al (Δ*G* ^≠^∼40.4 kJ mol^−1^ at 298 K, see above) or for aggregation steps only an entropic barrier, analogous to that for dimerization of Me_3_Al (ca. 31.8 kJ mol^−1^ at 298 K based on experiment).

A second, analogous step between **3,3** and **1,2** provides chain **4,5** which can rearrange to **4,3**, the smallest 5‐coordinate sheet located by theory, accompanied by successive losses of Me_3_Al. Overall, this assembly process is exergonic by 31.5 kJ mol^−1^ but only because the Me_3_Al that is produced can dimerize to form Me_6_Al_2_. Otherwise, the process is thermodynamically uphill (blue curve) but could be driven to completion by competing hydrolysis of Me_3_Al.

As the elementary steps do involve loss of Me_3_Al, the overall rate of this complex aggregation process would be dictated by the highest energy transition state (ca. 90 kJ mol^−1^) along the entire pathway. For a unimolecular process this would correspond to a reaction with a half‐life of ca. 15 min at room temperature. This is on the same time scale as the monitoring experiments and given that bimolecular processes are often accelerated in solution the process seems reasonable.

An anion [**4,3**]^−^ (*m/z*=463) derived from **4,3** or **4,4** was seen in these monitoring experiments.^[23]^ We do note that sheet **4,3** is also accessible in fewer steps through dimerization of **2,3**, forming **4,5**+Me_3_Al. Indeed, when this process is included, one would predict that sheet **4,3** would be the major aggregation product formed from a mixture of **1,3** and **2,4** formed via hydrolysis.

Numerical simulation of this process was investigated using the energies and barriers determined by theory and details are provided as Supporting Information (Table S4, Figure S7). The kinetic product distribution (i. e. the ratio of **4,m** vs. **3,m** species) is sensitive to the Al : O stoichiometry. This type of behaviour was seen in the monitoring experiments (i. e. variations in even vs. odd anion intensity with Al : O stoichiometry), though not systematically explored.^[23]^

Ions [**7,4**]^−^ or [**8,4**]^−^ are the first intense ions seen in monitoring experiments in PhF. Their normalized intensities vs. time exhibit behaviour expected for intermediates involved in consecutive, reactions, with their formation being about 10× faster than their disappearance (Figure S4a). Since we cannot derive actual concentrations from this intensity data, there is little point in trying to fit this data to an underlying mechanism. However, the intensity data can be fit to consecutive 1^st^ order reactions for which analytical solutions are well known. The formation processes involve rates equivalent to those for a unimolecular process with a barriers of about 80–90 kJ mol^−1^ at 298 K. This is similar to that estimated by theory for aggregation to form **4,3**.

The formation of larger sheets becomes increasingly exergonic as the number of dative Al−O interactions increase in the products. For sheets **5,4 6,5** and **7,5** the transformation features Δ*G*=−30 to −69 kJ mol^−1^ with respect to **4,3** depending on whether **1,3** vs. **2,4** is involved. In fact, the overall free energy changes effectively mean growth by this mechanism would be irreversible. Further, it should be borne in mind that Me_3_Al, which is generated during the individual steps, is being rapidly (and irreversibly) consumed through hydrolysis and termination reactions involving intermediates analogous to those shown in Scheme 2.

Another feature of the experimental growth reaction is that under certain conditions, low MW anion precursors are transforming directly to higher MW precursors without the detectable intervention of intermediates. These observations suggest it might also be possible for larger sheets to fuse together at some point in the growth process, thus by‐passing intermediates that can only form in a step‐wise manner involving low MW oligomers like **1,3** or **2,4**. Simplistically, **7,4**+**7,4** might give the precursors to [**14,5**]^−^ or [**14,6**]^−^ via direct reaction, accompanied by loss of Me_6_Al_2_.

**Figure 5:**
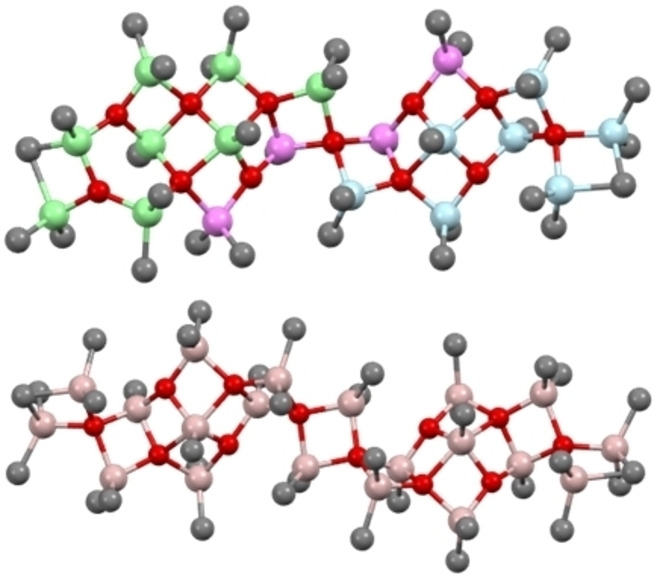
Structure of 5‐coordinate sheet **12,7** built up from **4,3** (blue), **5,3** (green) sheets and the Sinn tetramer **3,1** (violet); Sheet **12,8** ‐ a dimer of two **6,4** sheets.

This concept finds some support in the structures of the larger 5‐C sheets[^23^, ^24^] – for example there are two sheet structures with composition **12,7** which differ in energy by 3.4 kJ mol^−1^. The more stable 5‐C sheet might have formed by fusing the Sinn tetramer (or a saturated version thereof) with saturated versions of **4,3** and **5,3** (Figure 5). Moreover, another stable sheet in this size range is **12,8** which features two **6,4** sheets fused to one another. It is anticipated that formation of **12,8** from **6,4** would proceed without an electronic barrier (analogous to the dimerization of Me_3_Al) but we have not investigated these reactions as we are uncertain as to which combinations give rise to which larger sheets.

## Simulation of the Growth Reaction

We have suggested that there are at least three growth phases ‐ assembly of small sheets from linear precursors, possible fusion of small sheets into larger ones, all of which at a certain size range become detectable by ESI‐MS, and a slower growth phase, related to aging, that leads to maturation of the larger sheets which have a local minimum in stability with **16,6**. To model this complex process, we made several simplifying assumptions, based on the theory just presented.

First, water is consumed through rapid formation of the Sinn dimer and trimer, stabilized by binding of Me_3_Al where the stable forms **1,3** and **2,4** located by theory are favoured at equilibrium. We used a simplified set of reactions to represent this process rather than the detailed simulation discussed earlier.

$$\mathrm{Me}{}_{6}\mathrm{Al}{}_{2}+H{}_{2}O\to 1,1+2\;\mathrm{CH}{}_{4}$$

$$1,1+\mathrm{Me}{}_{6}\mathrm{Al}{}_{2}\to 1,3$$

$$1,3+H{}_{2}O\to 2,2+2\;\mathrm{CH}{}_{4}$$

$$2,2+\mathrm{Me}{}_{6}\mathrm{Al}{}_{2}\to 2,4$$

The two hydrolysis steps were rate determining with a rate constant set to 1200 M^−1^ min^−1^ as this roughly corresponds to the overall rate constant for this multi‐step process. Similarly, binding of Me_3_Al to the products was simplified to one step with a rate constant of 8800 M^−1^ min^−1^. In both cases binding is very favourable (see earlier discussion) and was rendered irreversible for convenience. This makes no difference to the simulation results within limits.

Second, these linear oligomers associate to form more stable aluminoxanes as was demonstrated in Figure 4, though again, a simplified set of equations was employed:

21,2→2,4

$$1,2+2,3\to 3,4+\mathrm{Me}{}_{3}\mathrm{Al}$$

$$2\;2,3\to 4,4+\mathrm{Me}{}_{6}\mathrm{Al}{}_{2}$$

Here is where we introduced a kinetic bias between odd‐ vs. even‐numbered species, where the fastest process (the third) proceeded 4 times more quickly than the slowest (the first). The fastest process had a rate constant of 8800 M^−1^ min^−1^. This may seem artificial but in the actual simulation (Supporting Information Figure S7) the **4,m** sheets were always favoured upon completion, and even during the initial kinetic stages.

We then allowed **3,4** and **4,4** to combine so as to produce a mixture of **6,4**, **7,4** and **8,4** (+ 2 Me_6_Al_2_) at identical rates (*k*=2200 M^−1^ min^−1^). Then, these intermediate sheets were combined to produce **12,6**‐**16,6** (+ Me_6_Al_2_) as suggested earlier but at 2‐fold lower rates (*k*=1100 M^−1^ min^−1^) compared with the slowest initial aggregation steps. This is largely based on the intuitive expectation that more steps are required for these fusion processes, and also by comparison of simulated to the experimental data, particularly the increase in average *m/z* ratio with time (Supporting Information, Table S5 and Figure S8).

Finally, we invoked “aging” of the mixture through reaction of these product sheets with **1,2** or **2,3** at the slowest rates (*k*=250 or 500 M^−1^ min^−1^). We retained a kinetic bias between reactions involving **1,2** (slowest) vs. **2,3** (faster). In order to reproduce the basic features seen we also required that conversion of **16,6** to higher MW sheets was 20× slower than conversion of lower MW sheets to **16,6**. This is consistent with a sheet of this size being a local minimum in stability.

The final part of the simulation deals with solution concentrations vs. anion intensities. In order to model the latter, we used the results of theory discussed in Ref. [24] (where [**7,4**]^−^ is the most stable anion per aluminoxane repeat unit) to weight the solution concentrations resulting from simulation. Full details are provided as Supporting Information.

**Figure 6:**
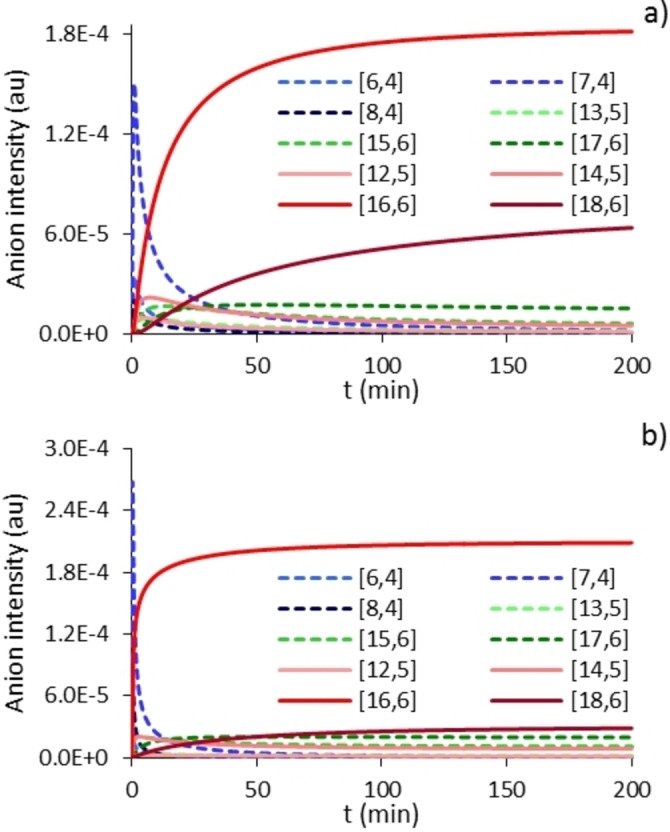
Individual anion intensities vs. time for simulated growth of activator precursors: a) Me_3_Al:H_2_O=4.4 : 1 b) .Me_3_Al:H_2_O=2.2 : 1.

Shown in Figure 6 are the anion intensities seen at two different Al : O ratios. We chose the latter so that Me_3_Al was always in local excess over dissolved water. Based on the earlier observation that the NMR spectra of these mixtures, upon completion, revealed a mixture of Me_3_Al and low MW aluminoxane,^[23]^ this must have been true for the monitoring reactions.

It is interesting to note that we are able to reproduce some of the experimental features of the growth reaction where the relative intensity of the low MW anions [**6,4**]^−^ to [**8,4**]^−^ appears sensitive to stoichiometry (Supporting Information, Figure S9), heightened amounts of [**18,6**]^−^ are present using larger Al : O ratios and the reaction to form [**16,6**]^−^ is actually *slower* at higher Al : O ratios. The finding that even‐numbered anions are favoured over odd‐numbered ones upon completion of the growth reaction is related to the initial assumptions about the reactivity of **2,3** vs. **1,2** as outlined in Scheme 4 and Figure 4. As we did not consider neutral stability in the aggregation or aging steps involving larger sheets (all occurring at the same rates except for those involving **16,6**), it is likely a more sophisticated simulation would capture the observed phenomena more accurately.

## Conclusion

Density functional theory, combined with numerical simulation provides a framework for the interpretation of the oligomerization reactions studied by ESI‐MS. Namely that hydrolysis of excess Me_3_Al under controlled conditions will produce a mixture of low MW, mainly linear aluminoxane precursors. These can aggregate into sheets, where the latter can grow either via the aging process involving addition of a low MW oligomer, or by mutual reaction along reactive edges. Cessation of growth by this process favors the formation of even‐numbered sheets as is observed experimentally.

As both theory and experiment concur that sheet structures are the reactive components in the size range of MAO suggested by experiment, it is of interest as to how for example metallocene catalyst activation occurs by MAO. We view the details of this process as still an open question, especially considering that the theoretical models for MAO employed to study this in the past were cages.[^14^, ^19^, ^21^] Future theoretical and experimental work should focus on this issue.

## Experimental Section

Normalized ESI‐MS intensity data (experiments originally reported in Ref. [22] and 23) are included as Supporting Information (Figures S3 to S6). Anion intensities were calculated using anion stability data reported in ref. 24 in PhF solvent using the Boltzmann distribution. These values were then used to weight the solution concentrations obtained by numerical simulation of the growth reaction using the COPASI software.^[35]^ The results are summarized in Figures 1, 2 and 6 while further details are reported as Supporting Information.

Density functional theory calculations were carried out by Gaussian 16 software,^[34]^ using the M06‐2X metahybrid GGA functional of the Minnesota series^[32]^ combined with the def‐TZVP basis set by Ahlrichs et al.^[33]^ Harmonic vibrational frequencies were calculated to confirm the structures either as a true minimum or a transition state in the potential energy surface. Gas phase Gibbs free energies were calculated at *T*=298 K and *p*=1 atm.

## Conflict of interest

The authors declare no conflict of interest.

## Supporting information

As a service to our authors and readers, this journal provides supporting information supplied by the authors. Such materials are peer reviewed and may be re‐organized for online delivery, but are not copy‐edited or typeset. Technical support issues arising from supporting information (other than missing files) should be addressed to the authors.

Supporting Information
