# Supplementary material for: Formation and Structure of Hydrolytic Methylaluminoxane Activators
Source: Chemistry. 2021 Oct 6;27(62):15460–71. doi: 10.1002/chem.202102463 (PMC8596698; doi:10.1002/chem.202102463)
Supplement: Supplementary file 1 — Supporting Information [file CHEM-27-15460-s001.pdf]

Chemistry–A European Journal

Supporting Information

Formation and Structure of Hydrolytic Methylaluminoxane Activators

Chemistry–A European Journal

Supporting Information

Formation and Structure of Hydrolytic Methylaluminoxane Activators

Table of Contents

Barriers to Dissociation of Me_6Al_2 and (MeAlO)(Me_3Al)$_3$ 2

Table S-1 Energies vs. Al-Al separation for Me_6Al_2 and **1,3** dissociating to form Me_3Al and **1,2** + Me_3Al at 298 K. 2

Re-analysis of Me_6Al_2 Methyl Group Exchange Data 2

Table S-2 NMR line shape data from ref. [30] 2

Simulation of Initial Hydrolysis of Me_6Al_2 2

Figure S-1 Simulation of Me_3Al hydrolysis invoking competing hydrolysis of Me_3Al vs. Me_6Al_2. Left: [H_2O] = 2 [Me_6Al_2] = 0.0275 M, Right: [H_2O] = [Me_6Al_2] = 0.055 M. 3

Simulation of Hydrolysis Network – Schemes 2 and 3 3

Table S-3 Reactions, barriers and free energy changes for network of reactions depicted in Schemes 2-3[a] 3

Figure S-2 Simulation of hydrolysis network, omitting formation of **2,0,2** and **3,1,1** from **2,1,1** and **3,2**, respectively. a) Al:O 1.5:1 b) Al:O 2:1 c) Al:O 3:1. 5

Summary of Previous Experimental Results 5

Figure S-3 Total ion counts (normalized to the most intense signal for each trace) vs. time for four different experiments involving hydrolysis of Me_3Al as monitored by ESI-MS.[22,23] 6

Figure S-4 a) Normalized ion intensities for selected individual ions [**n,m**]$^-$ vs. time with [H_2O] = 0.032 M, and [Me_3Al] = 0.038 in PhF in the presence of 0.2 mol% OMTS. b) Normalized, grouped ion intensities vs. time for the same experiment. 7

Figure S-5 a) Grouped ion intensities in PhF. Conditions [Me_3Al] = 0.16 M, [H_2O] = 0.032 M. In this case, the mass spectra are affected by in-source fragmentation,[23] so individual ions related by 72 Da (Me_3Al) have been grouped together. b) Grouped ion intensities in DFB. Conditions [Me_3Al] = 0.064 M, [H_2O] = 0.055 M. 7

Figure S-6 Mass spectra of MAO anions in a) PhF and b) DFB following completion of the growth reaction. Reproduced from ref. [23]. 8

Estimation of rate constants for formation and disappearance of [n,4]$^-$ anions. 8

Simulation of Aggregation. 9

Table S-4 – Reaction sequence corresponding to Figure 4. 9

Figure S-7 - Oligomer product distribution vs. time for hydrolysis of Me_3Al and oligomerization of **1,3** and **2,4** according to Figure 4. 10

Simulation of the Growth Reaction 11

Table S-5 Simulation of the Growth Reaction as depicted in Figure 6. 11

Figure S-8 – Average *m/z* ratio vs. time reported in ref. [22]. 11

Figure S-9 – Anion intensities vs. time based on numerical simulation and anion stabilities per repeat unit. ... 12

Structures and Energies 12

Table S-6 – Structures, electronic, free energy, enthalpy and entropy of transition states and products 12

Barriers to Dissociation of Me_6Al_2 and $(MeAlO)(Me_3Al)_3$

The electronic and free energies of both Me_6Al_2 and $(MeAlO)(Me_3Al)_3$ were monitored while increasing the Al-Al separation with the results shown in the Table below. A stationary point in which E was maximal with one imaginary frequency could not be located in either case, though plots of Δ*G* vs. Al-Al do show a maximum at ca. 40 kJ mol^{-1} in both cases.

Table S-1 Energies vs. Al-Al separation for Me_6Al_2 and **1,3** dissociating to form Me_3Al and **1,2** + Me_3Al at 298 K.

Me_6Al_2	*E* (au)	*H* (au)	*G* (au)	Δ*E* (kJ mol^{-1})	Δ*H* (kJ mol^{-1})	Δ*G* (kJ mol^{-1})
Al-Al_{eq} (270 pm)	-724.3129062	-724.077658	-724.137079			
290	-724.3039808	-724.070648	-724.130793	23.4	18.4	16.5
300	-724.3003146	-724.066911	-724.127206	33.1	28.2	25.9
310	-724.2974231	-724.064134	-724.125208	40.7	35.5	31.2
320	-724.2953673	-724.061989	-724.123512	46.0	41.1	35.6
330	-724.2940672	-724.060599	-724.122672	49.5	44.8	37.8
340	-724.2932374	-724.059819	-724.122495	51.6	46.8	38.3
350	-724.2926465	-724.059234	-724.121658	53.2	48.4	40.5
360	-724.2921387	-724.058763	-724.121682	54.5	49.6	40.4
370	-724.2916617	-724.057274	-724.123605	55.8	53.5	35.4
$(Me_2Al)_4(\mu^4\text{-}O)(\mu\text{-}Me)_2$						
Al-Al_{eq} (270 pm)	-1444.212552		-1443.895653			
290	-1444.207819		-1443.893149	12.4		6.6
300	-1444.202993		-1443.887331	25.1		21.8
310	-1444.198381		-1443.883062	37.2		33.1
320	-1444.19508		-1443.880129	45.9		40.8
330	-1444.194724		-1443.880315	46.8		40.3

Re-analysis of Me_6Al_2 Methyl Group Exchange Data

The rate constants for exchange of terminal and bridging Me groups in Me_6Al_2 have been measured by NMR and by several different authors. We used the data reported in ref. [30] which is reproduced in the Table below:

Table S-2 NMR line shape data from ref. [30]

T (°C)	*T* (K)	1/*T*	k_C (sec^{-1})	k_T (sec^{-1})	k_Ch/k*T*	k_Th/k*T*	ln(k_Ch/k*T*)	ln(k_Th/k*T*)
-55	218.15	4.58E-03	12.3	7.7	2.71E-12	1.68E-12	-26.6	-27.1
-50	223.15	4.48E-03	26.3	18.8	5.66E-12	4.04E-12	-25.9	-26.2
-45	228.15	4.38E-03	52.5	46.2	1.10E-11	9.72E-12	-25.2	-25.4

Plots of ln(k_ih/k*T*) vs. 1/*T* were linear in both toluene (T) and cyclopentane (C) solvents over this narrow temperature range. The following activation parameters were obtained: in toluene $\Delta H^‡$= 58.3 kJ mol^{-1} with $\Delta S^‡$ = 45.5 J mol^{-1} K^{-1} while in cyclopentane $\Delta H^‡$= 72.7 kJ mol^{-1} with $\Delta S^‡$ = 107.2 J mol^{-1} K^{-1} so that $\Delta G^‡$= 72.7 – (0.1072*298) = 40.7 kJ mol^{-1} at 298 K. The different values obtained in aromatic vs. aliphatic solvents have been attributed to participation of the aromatic solvent, and even different mechanisms. A full discussion of this early work is given in ref. [27].

Simulation of Initial Hydrolysis of Me_6Al_2

As mentioned in the manuscript, two possible pathways for hydrolysis of Me_6Al_2 involve $Me_3Al\text{-}OH_2$ vs. $Me_6Al_2\text{-}OH_2$ as intermediates. In the first case, formation of $Me_3Al\text{-}OH_2$ from Me_3Al and water is favourable but reversible and decomposition to form Me_2AlOH is rate-determining. The overall rate constant for decomposition is 2.08×10^7 M^{-1} s^{-1} based on the calculated barrier for the second step, assuming the first step (binding of water) is diffusion controlled (7.56×10^9 M^{-1} s^{-1}) but reversible. In the case of the second process, it is the first step which is rate-determining with an overall 2nd order rate constant of 570 M^{-1} s^{-1} and produces Me_5Al_2OH as the final product. The amounts of Me_6Al_2 and Me_3Al are

governed by the dissociation equilibrium, which is unfavourable but occurs at forward rate of 5×10^5 s^{-1} at room temperature with a reverse rate which is diffusion controlled. These values (each divided through by 100 for convenience) were used as input for the COPASI software with the results summarized below for a stoichiometric vs. two-fold excess of Me_6Al_2 over H_2O. We assumed all of the Me_6Al_2 was initially present as that material. It can be seen that bimolecular hydrolysis is not important under any conditions.

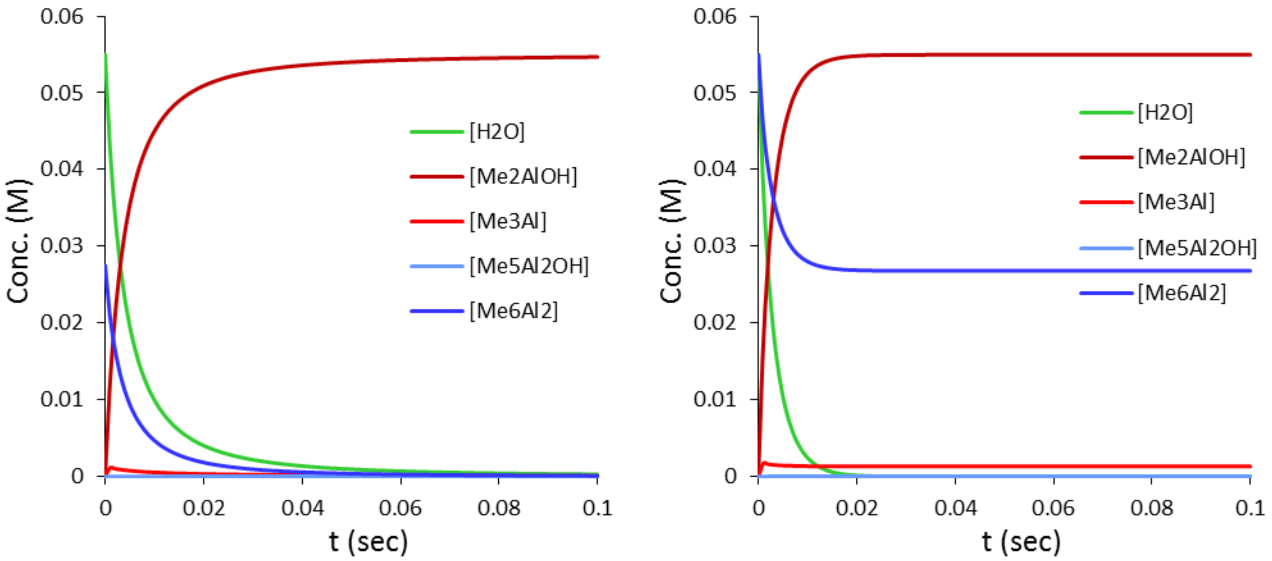

Figure S-1 Simulation of Me_3Al hydrolysis invoking competing hydrolysis of Me_3Al vs. Me_6Al_2. Left: $[H_2O] = 2 [Me_6Al_2] = 0.0275$ M, Right: $[H_2O] = [Me_6Al_2] = 0.055$ M.

Simulation of Hydrolysis Network – Schemes 2 and 3

This is described in some detail in the manuscript. The elementary steps involved are summarized in Table S-3 while simulations without, and with bimolecular condensation of $(Me_2AlOH)_2$, and Me_5Al_2OH are presented in Figures 1 and 2, respectively.

Table S-3 Reactions, barriers and free energy changes for network of reactions depicted in Schemes 2-3[a]

**Reaction**	**$\Delta G^‡$ / kJ mol^{-1}**	**ΔG / kJ mol^{-1}**
(1) $Me_6Al_2 + H_2O \rightarrow Me_6Al\text{-}OH_2$	62.8	7.2
(2) $Me_6Al_2\text{-}OH_2 \rightarrow Me_5Al_2(\mu\text{-}OH) + CH_4$	20.0	-223.9
(3) $Me_6Al_2\text{-}OH_2 \rightarrow Me_3Al\text{-}OH_2 + ½Me_6Al_2$	-	-28.3
(4) $Me_3Al\text{-}OH_2 \rightarrow Me_2AlOH$	60.3	-111.3
(5) $2Me_2AlOH \rightarrow Me_2Al(\mu\text{-}OH)_2AlMe_2$	-	-188.5
(6) $Me_5Al_2(\mu\text{-}OH) \rightarrow$ **1,1** + CH_4	107.9	-69.6
(7) $Me_5Al_2(\mu\text{-}OH) + ½Me_6Al_2 \rightarrow$ **1,2** + CH_4	42.1	-153.4
(8) $Me_5Al_2(\mu\text{-}OH) + H_2O \rightarrow Me_5Al_2(\mu\text{-}OH)\text{-}H_2O$	53.7	-31.1
(9) $Me_5Al_2(\mu\text{-}OH)\text{-}H_2O \rightarrow Me_2Al(\mu\text{-}OH)_2AlMe_2 + CH_4$	5.6	-205.6
(10) $Me_2Al(\mu\text{-}OH)_2AlMe_2 + H_2O \rightarrow Me_2Al(\mu\text{-}OH)_2AlMe_2\text{-}H_2O$	-	4.9
(11) $Me_2Al(\mu\text{-}OH)_2AlMe_2\text{-}H_2O \rightarrow Me_2Al(\mu\text{-}OH)_2Al(OH)Me + CH_4$	71.3	-142.0
(12) $Me_2Al(\mu\text{-}OH)_2Al(OH)Me + H_2O \rightarrow Me_2Al(\mu\text{-}OH)_2Al(OH)Me\text{-}H_2O$	-	3.4
(13) $Me_2Al(\mu\text{-}OH)_2Al(OH)Me\text{-}H_2O \rightarrow (OH)MeAl(\mu\text{-}OH)_2Al(OH)Me + CH_4$	71.8	-140.9
(14) $(OH)MeAl(\mu\text{-}OH)_2Al(OH)Me + H_2O \rightarrow (OH)MeAl(\mu\text{-}OH)_2Al(OH)Me\text{-}H_2O$	-	-12.1
(15) $(OH)MeAl(\mu\text{-}OH)_2Al(OH)Me\text{-}H_2O \rightarrow (OH)MeAl(\mu\text{-}OH)_2Al(OH)_2 + CH_4$	82.8	-120.5
(16) $(OH)MeAl(\mu\text{-}OH)_2Al(OH)_2 + H_2O \rightarrow (OH)MeAl(\mu\text{-}OH)_2Al(OH)_2\text{-}H_2O$	-	-12.7
(17) $(OH)MeAl(\mu\text{-}OH)_2Al(OH)Me\text{-}H_2O \rightarrow (OH)_2Al(\mu\text{-}OH)_2Al(OH)_2 + CH_4$	84.0	-118.7

(18) $Me_2Al(\mu\text{-}OH)_2AlMe_2$ + ½Me_6Al_2 → **1,1,1** + CH_4	45.6	-145.1
(19) $Me_2Al(\mu\text{-}OH)_2Al(OH)Me$ + ½Me_6Al_2 → **1,0,2** + CH_4	42.4	-201.8
(20) $(OH)MeAl(\mu\text{-}OH)_2Al(OH)Me$ + ½Me_6Al_2 → **1,0,1**-OH + CH_4	41.2	-206.5
(21) **1,2** + H_2O → **1,2**-H_2O	-	-54.6
(22) **1,2**-H_2O → **1,1,1** + CH_4	34.9	-173.7
(23) **1,1,1** + H_2O → **1,1,1**-H_2O	-	-57.1
(24) **1,1,1**-H_2O → **1,0,2** + CH_4	45.9	-136.6
(25) **1,0,2** + H_2O → **1,0,2**-H_2O	-	-36.2
(26) **1,0,2**-H_2O → **1,0,1**-OH + CH_4	55.7	-106.0
(27) **1,1,1** + ½Me_6Al_2 → **2,2** + CH_4	42.0	-151.5
(28) **1,0,2** + ½Me_6Al_2 → **2,1,1** + CH_4	42.1	-148.5
(29) **1,0,1**-OH + ½Me_6Al_2 → **2,0,2** + CH_4	42.6	-216.7
(30) **2,2** + H_2O → **2,2**-H_2O	-	-55.1
(31) **2,2**-H_2O → **2,1,1** + CH_4	48.0	-135.5
(32) **2,1,1** + H_2O → **2,1,1**-H_2O (ladder)	-	-59.1
(33) **2,1,1** + H_2O → **2,1,1**-H_2O (cage)	-	-60.4
(34) **2,1,1**-H_2O → **2,0,2** + CH_4 (ladder)	31.6	-151.3
(35) **2,1,1**-H_2O → **2,0,2** + CH_4 (cage)	36.4	-160.4
(36) **2,1,1** + ½Me_6Al_2 → **3,2** + CH_4	44.3	-145.2
(37) **2,0,2** + ½Me_6Al_2 → **3,1,1** + CH_4 (ladder)	44.7	-149.1
(38) **2,0,2** + ½Me_6Al_2 → **3,1,1** + CH_4 (cage)	47.5	-163.4
(39) **3,2** + H_2O → **3,2**-H_2O	-	-42.0
(40) **3,2**-H_2O → **3,1,1** + CH_4 (sheet)	7.4	-217.5
(41) **3,1,1** + ½Me_6Al_2 → **4,2** + CH_4 (ladder)	46.4	-145.6
(42) **3,1,1** + ½Me_6Al_2 → **4,2** + CH_4 (cage)	50.8	-158.3
(43) **3,1,1** + ½Me_6Al_2 → **4,2** + CH_4 (sheet)	64.3	-145.9
(44) Me_5Al_2OH + Me_6Al_2 → $Me_5Al_2OH\text{-}Me_6Al_2$	-	25.7
(45) $Me_5Al_2OH\text{-}Me_6Al_2$ → $Me_5Al_2OH\text{-}Me_3Al$ + ½Me_6Al_2	57.9	10.4
(46) $Me_2Al(\mu\text{-}OH)_2AlMe_2$ + Me_6Al_2 → $Me_2Al(\mu\text{-}OH)_2AlMe_2\text{-}Me_6Al_2$	-	29.3
(47) $Me_2Al(\mu\text{-}OH)_2AlMe_2$ → $Me_2Al(\mu\text{-}OH)_2AlMe_2\text{-}Me_3Al$ + ½Me_6Al_2	60.2	10.9
(48) Me_5Al_2OH + Me_6Al_2 → **1,3** + CH_4 (local minimum)	110.8	-162.2
(49) $Me_2Al(\mu\text{-}OH)_2AlMe_2$ + Me_6Al_2 → **1,2,1** + CH_4	113.3	-160.3
(50) $2Me_2Al(\mu\text{-}OH)_2AlMe_2$ → $Me_7Al_4(OH)_3$ + CH_4	78.1	-145.1
(51) $Me_7Al_4(OH)_3$ → **2,0,2** + CH_4 (ladder)	87.2	-100.4
(52) $Me_7Al_4(OH)_3$ → **2,0,2** + CH_4 (cage)	101.9	-109.6
(53) **2,0,2** (ladder) → **2,0,2** (cage)	64.1	-9.3
(54) **2,0,2** (cage) → **3,0,1** + CH_4 (cage)	143.0	-52.4
(55) **3,0,1** (cage) → **4,0** + CH_4 (cage)	84.4	-87.4
(56) $Me_2Al(\mu\text{-}OH)_2AlMe_2$ + Me_5Al_2OH → $Me_8Al_4(OH)_2$ + CH_4 route A	87.1	-159.5
(57) $Me_2Al(\mu\text{-}OH)_2AlMe_2$ + Me_5Al_2OH → $Me_8Al_4(OH)_2$ + CH_4 route B	72.5	-162.7
(58) $Me_8Al_4(OH)_2$ → **2,1,1** + CH_4 route A	88.3	-111.2
(59) $Me_8Al_4(OH)_2$ → **2,1,1** + CH_4 route B	102.0	-108.0
(60) $2Me_5Al_2OH$ → Me_9Al_4OH + CH_4	80.5	-164.7
(61) $Me_8Al_4(OH)_2$ → i-**2,2** + CH_4	86.2	-112.8

a. Where no $\Delta G^{\ddagger}$ is reported, the reaction has no electronic barrier but will have an entropic one.

Higher MW aluminoxanes are being formed through hydrolysis of intermediates involved in the formation of lower MW aluminoxanes. For example, no **4,2** forms from $[MeAl(OH)_2]_2$ or even **1,0,1**-OH (Scheme 2); this is easy to show by simply

eliminating the relevant steps from the simulation. The final product of this network is being exclusively formed from **2,0,2** or **3,1,1** as is illustrated in Figure S-2 where hydrolyses leading to these species are omitted. Roughly half of the **4,2** that is produced is being formed by direct hydrolysis of **3,2**, while the remainder is derived from **2,0,2** which can form from hydrolysis of **2,1,1** or by bimolecular condensation of $(Me_2AlOH)_2$ (Scheme 3).

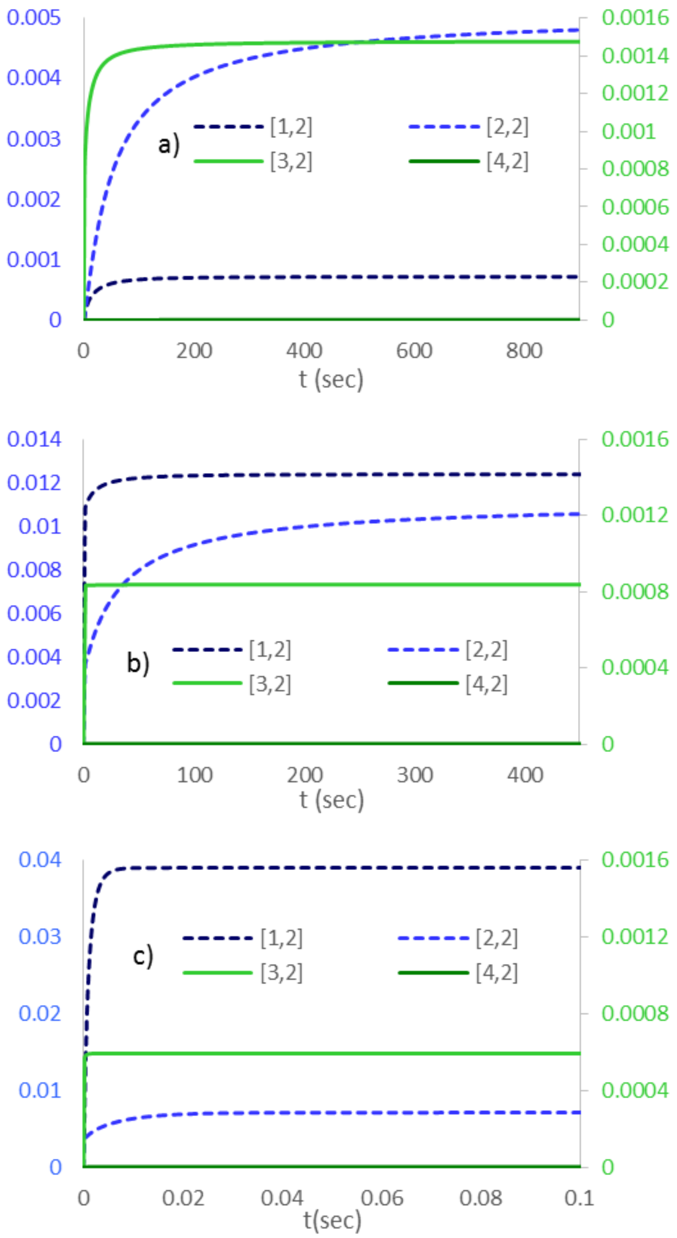

Figure S-2 Simulation of hydrolysis network, omitting formation of **2,0,2** and **3,1,1** from **2,1,1** and **3,2**, respectively. a) Al:O 1.5:1 b) Al:O 2:1 c) Al:O 3:1.

Concentrations of **3,2** and **4,2** ~ 0 for all traces are plotted in green using the right hand axis, while **1,2** and **2,2** are plotted in blue using the left hand ordinate. These can be compared to Figures 2b)-d).

Summary of Previous Experimental Results

The hydrolysis of Me_3Al as monitored by ESI-MS reported in ref. [**Error! Bookmark not defined.**] has been interpreted based upon a mechanism that invokes step-wise formation of MAO involving Me_2AlOH (or a related species) as an intermediate, coupled with aggregation of neutral aluminoxane precursors to form higher MW material. Based upon our analysis of the initial steps, Me_2AlOH is far too reactive a species to persist in the amounts needed to form high MW

aluminoxane, while stable forms $(Me_2AlOH)_n$ are not prone to dissociation of monomer and suffer other reactions, as outlined in the manuscript.

In subsequent work,[Error! Bookmark not defined.] the increase in the average *m/z* ratio of these mixtures with time was shown to feature at least two underlying rates, where the slower process seemed related to aging of MAO.[Error! Bookmark not defined.b] The initial stages of the aging process appears to involve step-wise homologation of a neutral aluminoxane $(MeAlO)_n(Me_3Al)_m$ to form $(MeAlO)_n(Me_3Al)_{m+1}$ through reaction with a low MW aluminoxane oligomer such as $Me_2AlOAlMe_2$ with loss of Me_3Al. Since this reaction can involve any neutral aluminoxane, it is likely that one mechanism for homologation of these species does involve this reaction. Evidently it is slow compared with any other underlying process though that could be due to very low background concentrations of these unstable, low MW oligomers.[Error! Bookmark not defined.]

Finally, it was obvious from the mass spectra recorded at different times in ref. [**Error! Bookmark not defined.**] that different ions (and their precursors) were appearing and disappearing at different rates, while the rates of these processes differed significantly in the two different solvents used under the conditions studied. Specifically, those anions with the composition $[(MeAlO)_n(Me_3Al)_mMe]^-$ (hereinafter $[\mathbf{n,m}]^-$) with m = 4, showed mainly decay behaviour, while those with m = 6 showed mainly growth behavior. Intermediate species with m = 5 were not analyzed in detail but their total intensity was a smaller fraction of the total ion intensity.

Unfortunately, the increase in total ion count (TIC) with time in these experiments, while consistent with an underlying increase in total ion concentration,[Error! Bookmark not defined.] is uncorrelated with experimental variables such as reactant concentrations, or changes in solvent at similar concentrations as illustrated in Figure S-3.

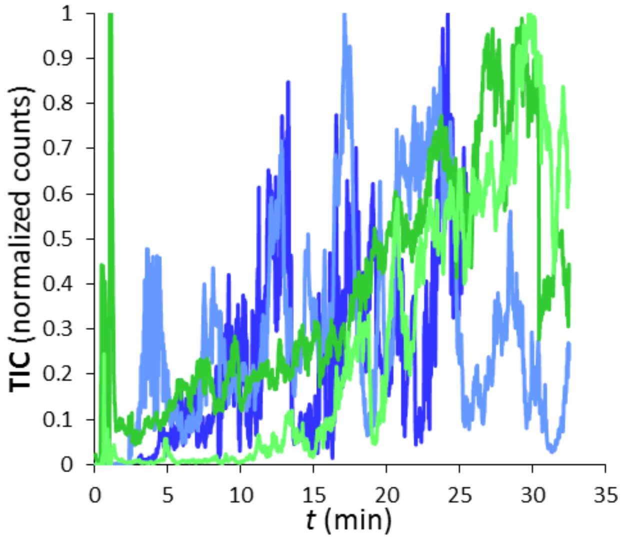

Figure S-3 Total ion counts (normalized to the most intense signal for each trace) vs. time for four different experiments involving hydrolysis of Me_3Al as monitored by ESI-MS.[22,23]

The light and dark green traces are for two experiments in DFB featuring $[Me_3Al]$ ~ $[H_2O]$ = 0.055 M, while the light and dark blue traces are for two experiments in PhF with $[H_2O]$ = 0.032 M and $[Me_3Al]$ = 0.16 and 0.038 M, respectively.

What does correlate better to experimental variables is the average *m/z* ratio,[Error! Bookmark not defined.] and also normalized ion intensities (i.e. individual ion counts divided through by the TIC for each time point). Shown in Figures S-4 and S-5 are the same experiments as depicted in Figure S-3.

In Figure S-4 we see results for individual (Figure S-4a) and grouped ion intensities (Figure S-4b) under near stoichiometric conditions in PhF. The basic trends confirm earlier observations – namely that the $[\mathbf{n,4}]^-$ (n = 6-10) are initially intense and decay while the higher MW $[\mathbf{n,m}]^-$ anions (n = 14-18, m = 5-6) show mainly growth characteristics. Ions intermediate in MW (n = 9-13, m = 5) show growth followed by decay. After 25 min at room temperature In PhF we see that $[\mathbf{14,m}]^-$ anions are the most intense, followed by $[\mathbf{15,6}]^-$ and $[\mathbf{16,6}]^-$, very different from what is seen over a similar time period in DFB (Figure S-5b). Note also that $[\mathbf{8,4}]^-$ is the most intense anion seen at short times, followed by $[\mathbf{7,4}]^-$.

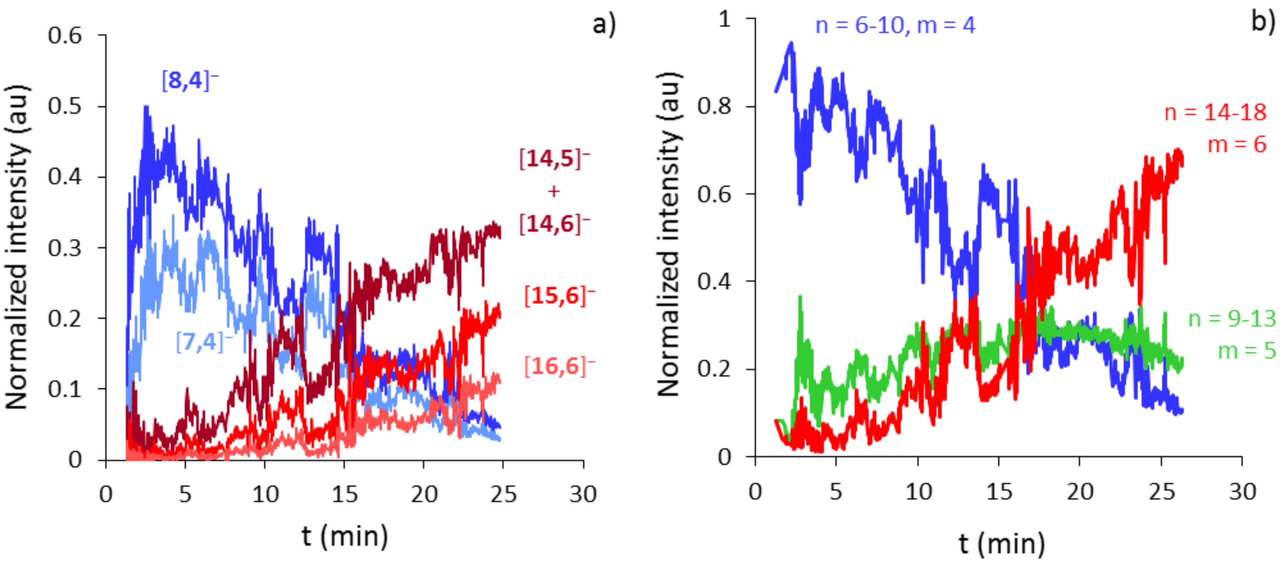

Figure S-4 a) Normalized ion intensities for selected individual ions [**n,m**]⁻ vs. time with $[H_2O]$ = 0.032 M, and $[Me_3Al]$ = 0.038 in PhF in the presence of 0.2 mol% OMTS. b) Normalized, grouped ion intensities vs. time for the same experiment.

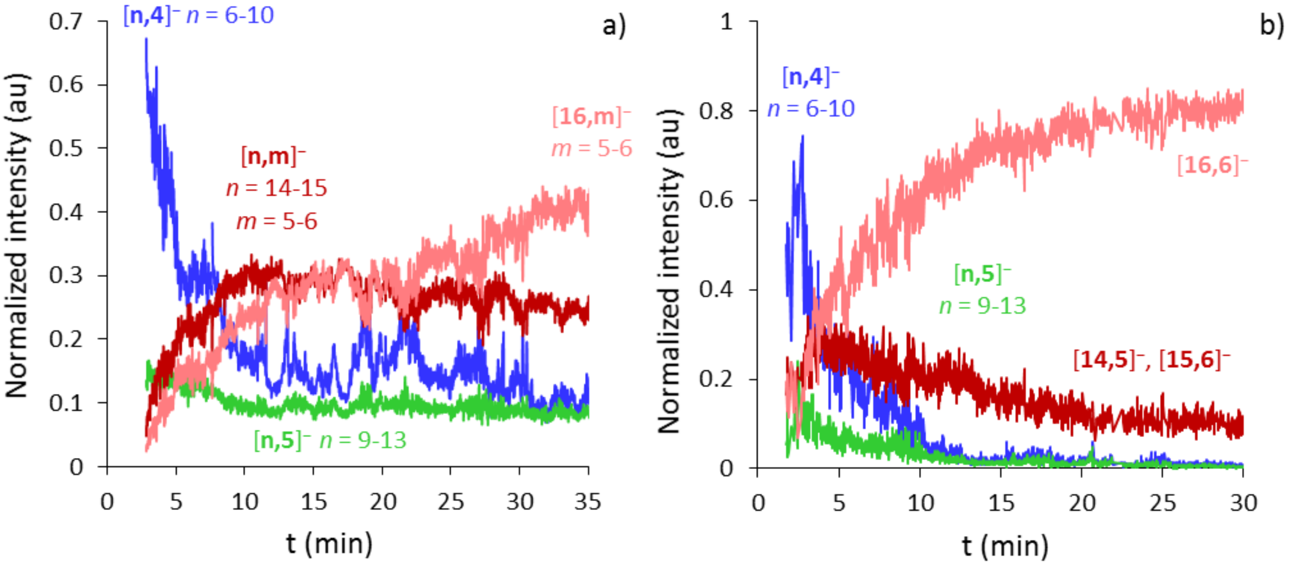

Figure S-5 a) Grouped ion intensities in PhF. Conditions $[Me_3Al]$ = 0.16 M, $[H_2O]$ = 0.032 M. In this case, the mass spectra are affected by in-source fragmentation,[Error! Bookmark not defined.] so individual ions related by 72 Da (Me_3Al) have been grouped together. b) Grouped ion intensities in DFB. Conditions $[Me_3Al]$ = 0.064 M, $[H_2O]$ = 0.055 M.

In Figure S-5a) we see data for an experiment featuring a 5-fold excess Me_3Al over water in PhF. Unlike the previous experiment, the mass spectra are complicated by minor oxidation (which impedes the growth process[Error! Bookmark not defined.]) and the appearance of additional ions related by ±72 Da, arising from in source fragmentation.[Error! Bookmark not defined.] Decay of the [**n,4**]⁻ anions is significantly faster, while the most intense *m* = 4 anion is [**7,4**]⁻ under these conditions at ca. 50% of the total intensity for this group of ions. The build-up of intensity for the major, higher MW anions (red traces) is initially rapid, though a slower process that results in formation of additional [**16,m**]⁻ (*m* = 5-6) is evident while the anions [**n,m**]⁻ (*n* = 14-15, *m* = 5-6) decay in intensity over the same time period. The [**n,5**]⁻ (*n* = 9-13) anions show mainly gradual decay under these conditions (green trace), while both these and the [**n,4**]⁻ anions persist in the mixture at longer times. Analysis of the average composition of this mixture (i.e. the average *m/z* ratio) shows that the degree of polymerization is significantly depressed (average *m/z* 1200 vs. 1330 Da) compared to experiments in DFB.[Error! Bookmark not defined.]

In the case of Figure S-5b), the solvent is DFB with higher moisture content [H_2O] = 0.055 M, and with [Me_3Al] = 0.062 M about 60% of the total reactant concentration used in Figure S-5a). Upon detection of signal, the decay of the [**n,4**]$^-$ anions to near zero is very rapid, as is the increase in [**16,6**]$^-$under these conditions. As mentioned elsewhere, the longer term growth in the latter anion is at the expense of high MW anions [**14,5**]$^-$ and especially [**15,6**]$^-$. The major [**n,4**]$^-$ anion detected is [**7,4**]$^-$ though significant amounts of [**9,4**]$^-$ are also present at short times.

Unfortunately, we cannot relate these changes to the underlying concentrations of neutral precursors because anion stabilities differ significantly between low vs. higher MW anions or even amongst ions similar in *m/z* ratio,[Error! Bookmark not defined.] while the normalized intensity data are not properly scaled to total ion concentration, whose time dependence is unknown in all experiments. In other words, the intensity of e.g. the [**n,4**]$^-$ anions, relative to the [**n,6**]$^-$ product ions is exaggerated by the process of normalization.

So, we relate our theoretical results and modeling of the growth reaction to the normalized intensities or measures of average composition such as the average *m/z* ratio recognizing that our main goal is to reproduce the temporal changes seen, or changes in ion speciation, rather than any quantitative changes based on an underlying mechanism for aluminoxane formation. The final feature not appreciated in earlier work is that the mass spectra of these mixtures, following completion of the growth reaction are dominated by even-numbered anions. For representative mass spectra see Figure S-6.

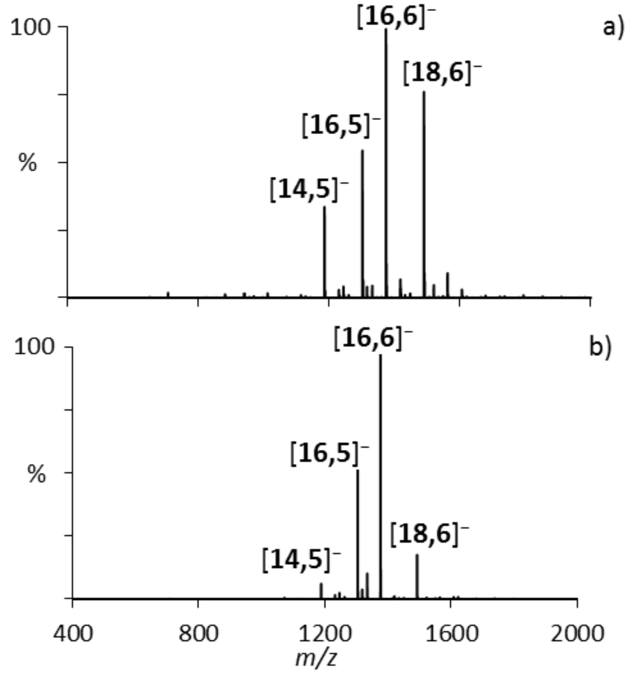

Figure S-6 Mass spectra of MAO anions in a) PhF and b) DFB following completion of the growth reaction. Reproduced from ref. [23].

Estimation of rate constants for formation and disappearance of [n,4]$^-$ anions.

The normalized intensity data shown in Figure S-4a cannot be converted into actual concentrations since total ion concentrations with time are not available from any of these experiments. However, the data for e.g. [**8,4**]$^-$ can be fit to the following equation y = a*[exp(-b*t)-exp(-c*t)] which is of the form expected for consecutive (1st order) reactions involving an intermediate. Non-linear least squares regression yields b = 1.038 and c = 0.0794 min^{-1} with R = 0.96.

The data which appears in Figure S-5b for the [**n,4**]$^-$ anions was analyzed in the same manner. However, in this case there was no data for the appearance of these ions (almost instantaneous upon acquisition of signal) so the only meaningful parameter was for disappearance of these ions with c ~ 0.303 min^{-1}. When corrected for the difference in total concentration, the value was 0.106 min^{-1} suggesting that the disappearance of these ions occurs at the same rate and by a similar mechanism in the two different solvents.

Since we do not know the actual concentration of these ions, it is not possible to extract actual rate constants, even if the underlying mechanism were known. However, these unimolecular rate constants correspond to barriers of 83 and 89 kJ mol^{-1} at 298 K suggesting the actual barriers for bimolecular formation and disappearance of the ion precursors are lower.

Simulation of Aggregation.

The processes involved in Figure 4 were used as input for COPASI using the energetics summarized in Table S-4. In addition, dimerization of **2,3** according to the following reactions (see Table S-4 for energies) was studied. In this Table and the equation below, the linear chains **n,m** are further classified as to the number of 4- vs. 3-coordinate oxygens in their structure. For example, in **4,6**-o4444, the global mínimum for this **n,m** all of the oxygens are 4-coordinate, while in the final product **4,5**-o3433, only the second O atom along the chain is 4-coordinate.

1) 2 **2,3** ↔ **4,6**-o4444 2) **4,6**-o4444 ↔ **4,5**-o4443 3) **4,5**-o4443 ↔ **4,5**-o4433 4) **4,5**-o4433 ↔ **4,5**-o3433

All steps were rendered reversible with the forward rate constants calculated from the barriers (calculated or estimated) and the Eyring relationship, while the reverse reactions were calculated from the free energy differences for each step. This simulation involved only a mixture of **1,3** and **2,4** to delineate basic features.

Table S-4 – Reaction sequence corresponding to Figure 4.

**Reaction [a]**	**$\Delta G^{\ddagger}$ / kJ mol^{-1} [b]**	**ΔG / kJ mol^{-1}**
(62) **1,2** + ½Me_6Al_2 → **1,3**	entropic	-13.7
(63) **1,2** + **1,2** → **2,4**	entropic	-38.1
(64) **2,4** → **2,3** + ½Me_6Al_2	entropic	15.8
(65) **1,2** + **2,3** → **3,5**-o444	entropic	-33.2
(66) **3,5**-o444 → **3,5**-o434	57.8	12.9
(67) **3,5**-0434 → **3,4**-o433	entropic	-12.9
(68) **3,4**-o433 → **3,3**-0333	entropic	14.7
(69) **3,3**-o333 + **1,2** → **4,5**-o3433	entropic	-21.7
(70) **4,5**-o3343 → **4,5**-o3333	50.0	17.4
(71) **4,5**-o3333 → **4,4**-o3333	entropic	-27.8
(72) **4,4**-o3333 → **4,4**-4C	39.4	-5.1
(73) **4,4**-4C → **4,3**-4C	entropic	8.3
(74) **4,3**-4C → **4,3**-5C	25.5	-27.2
(75) 2 **2,3** -> **4,6**-o4444	entropic	-33.3
(76) **4,6**-o4444 -> **4,5**-o4443	entropic	12.7
(77) **4,5**-o4443 -> **4,5**-o4433	33.0	-8.5
(78) **4,5**-o4433 -> **4,5**-o3433	49.3	11.2

[a] Entropic barriers are 31.8 kJ mol^{-1} for aggregation steps and 40.4 kJ mol^{-1} for loss of Me_3Al. The former have no electronic barrier while the latter were deemed equivalent to that for calculated for dissociation of Me_6Al_2.

In the figures which follow (Figure S-7), speciation has been simplified to **3,5**, **4,5**, **4,4** and **4,3** where the individual traces correspond to the *sum* of the concentrations of the various isomers present. In fact, in all cases because of their relative stability (Figure 4, Table S-4), the concentrations track the most stable isomer (**3,5**-o444, **4,5**-o4433, **4,4**-4C and **4,3**-5C, respectively).

The reversibility imposed leads to different behaviour at different time scales. In essence, a mixture of **3,5** and **4,5** + Me_6Al_2 forms rapidly (within msec) from **1,3** and **2,4** where **4,5** is being *exclusively* formed by the much shorter dimerization pathway involving **2,3**. These species are more gradually depleted of Me_3Al forming some **4,3**, **3,4** and even **3,3** on the time scale of seconds, along with Me_6Al_2.[a] The final phase involves reversible formation of **1,2** from these species, so that **3,4** and **3,3** are converted slowly to **4,3**, which based on its thermodynamic stability, is the ultimate product. This is

[a] If experimental data are used, dimerization of Me_3Al with $K \sim 1\times10^5$ M^{-1} at 298 K is the most favourable of the various binding events.

illustrated in Figure S-7 for two different amounts of **1,3** and **2,4**. The ratio of these two precursors has an influence on the initial (kinetic) product distribution, and rate of approach to equilibrium but obviously not on the overall outcome.

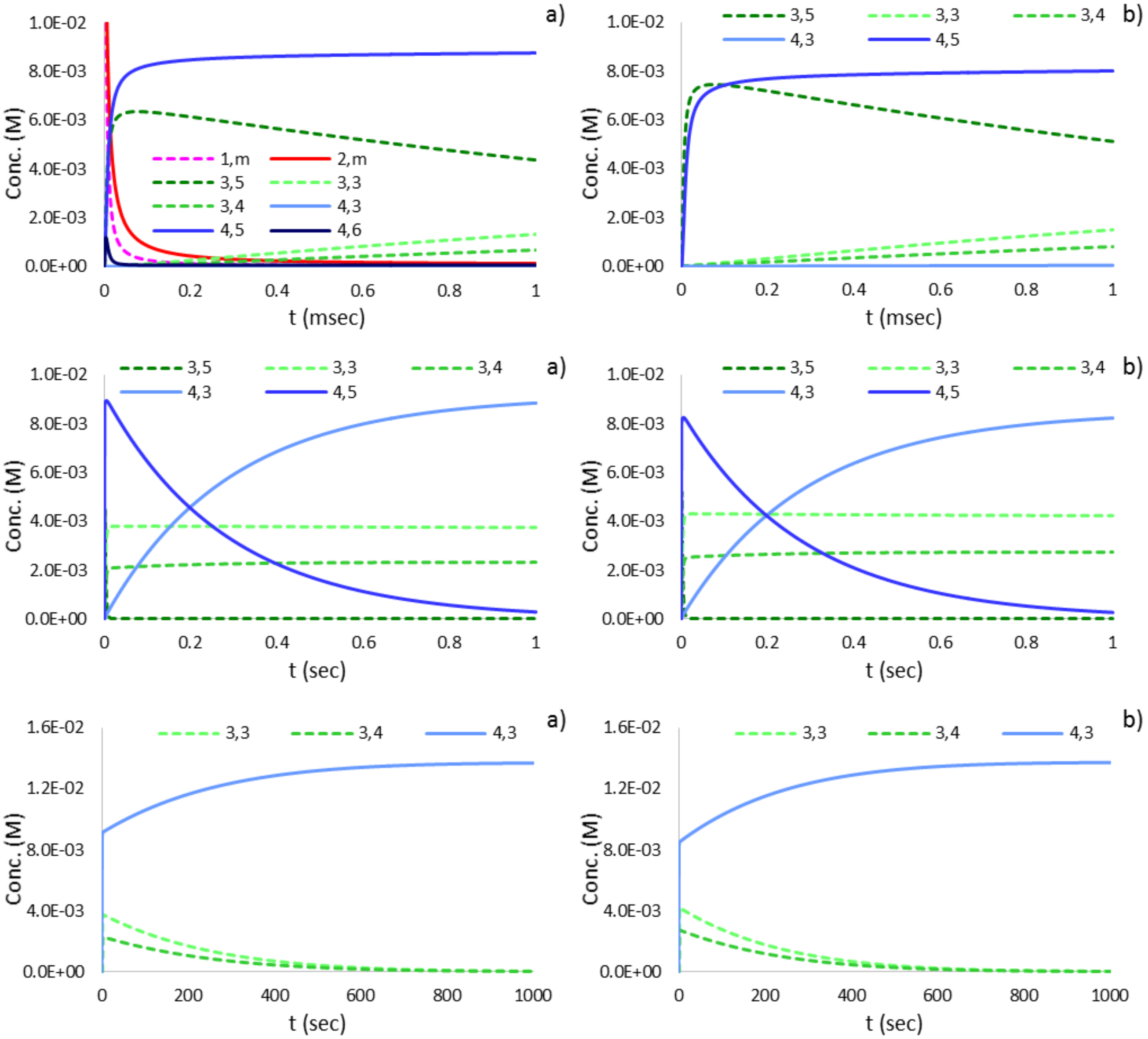

Figure S-7 - Oligomer product distribution vs. time for hydrolysis of Me_3Al and oligomerization of **1,3** and **2,4** according to Figure 4.

a) 1:1 mixture of **1,3** and **2,4** = 0.0183 M at three different time scales b) 2:1 mixture of **1,3** and **2,4** = 0.01375 M at the same time scales.

Simulation of the Growth Reaction

The basic features are summarized in the text and a table of reactions and rate constants used in the numerical simulation follow. These were chosen so that the average *m/z* ratio of the mixture changed in a manner very similar to that reported in ref. [22]. See Figure S-8 for an overlay of calculated and experimental data. Also shown in Figure S-9 are some individual anion intensities at short reaction times, corresponding to Figure 6 in the manuscript. Note the difference in the relative intensity of [**7,4**]$^-$ vs. [**8,4**]$^-$ and [**6,4**]$^-$. Similar effects were seen at different Al:O ratios in PhF as reported in ref. [23].

Table S-5 Simulation of the Growth Reaction as depicted in Figure 6.

Reaction	k_f (M^{-1} min^{-1}) or k_f (min^{-1})	k_r (M^{-1} min^{-1})	Reaction	k_f (M^{-1} min^{-1}) or k_f (min^{-1})
Me_6Al_2 = 2 * $AlMe_3$	8800	1.26e8	6,4 + 8,4 -> 14,6 + Me_6Al_2	1100
1,3 = 1,2 + $AlMe_3$	8800	1.26e8	2 7,4 -> 14,6 + Me_6Al_2	1100
2,4 = 2,3 + $AlMe_3$	8800	1.26e8	7,4 + 8,4 -> 15,6 + Me_6Al_2	1100
Me_6Al_2 + H_2O -> 1,1 + 2 * CH_4	1200		2 8,4 -> 16,6 + Me_6Al_2	1100
1,1 + Me_6Al_2 -> 1,2 + $AlMe_3$	8800		12,6 + 1,2 -> 13,6 + Me_6Al_2	250
1,3 + H_2O -> 2,2 + 2 * CH4	1200		13,6 + 1,2 -> 14,6 + Me_6Al_2	250
2,2 + Me_6Al_2 -> 2,4	8800		14,6 + 1,2 -> 15,6 + Me_6Al_2	250
2 * 2,3 -> 4,4 + Me_6Al_2	8800		15,6 + 1,2 -> 16,6 + Me_6Al_2	250
1,2 + 2,3 -> 3,4 + $AlMe_3$	4400		16,6 + 1,2 -> 17,6 + Me_6Al_2	12.5
2 * 1,2 -> 2,4	2200		17,6 + 1,2 -> 18,6 + Me_6Al_2	250
2 * 3,4 -> 6,4 + 2 * Me_6Al_2	2200		12,6 + 2,3 -> 14,6 + 1.5 Me_6Al_2	500
3,4 + 4,4 -> 7,4 + 2 * Me_6Al_2	2200		13,6 + 2,3 -> 15,6 + 1.5 Me_6Al_2	500
2 * 4,4 -> 8,4 + 2 * Me_6Al_2	2200		14,6 + 2,3 -> 16,6 + 1.5 Me_6Al_2	500
2 6,4 -> 12,6 + Me_6Al_2	1100		15,6 + 2,3 -> 17,6 + 1.5 Me_6Al_2	500
6,4 + 7,4 -> 13,6 + Me_6Al_2	1100		16,6 + 2,3 -> 18,6 + 1.5 Me_6Al_2	25

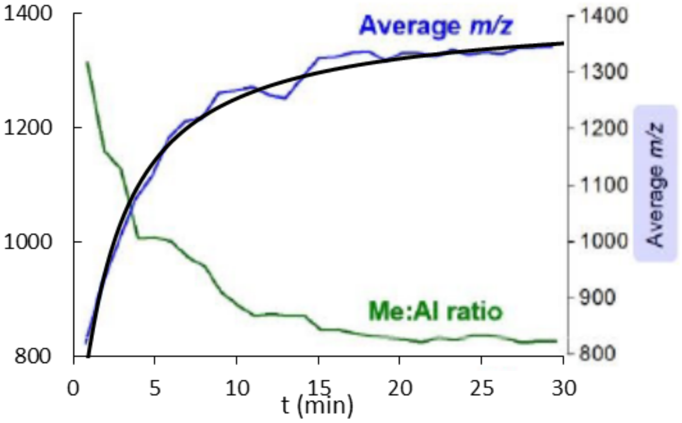

Figure S-8 – Average *m/z* ratio vs. time reported in ref. [22].

Experimental figure reproduced with permission from the authors. Overlay (black line) is the calculated *m/z* ratio of the mixture based on simulation weighted by anion stability per repeat unit (ref. [24]).

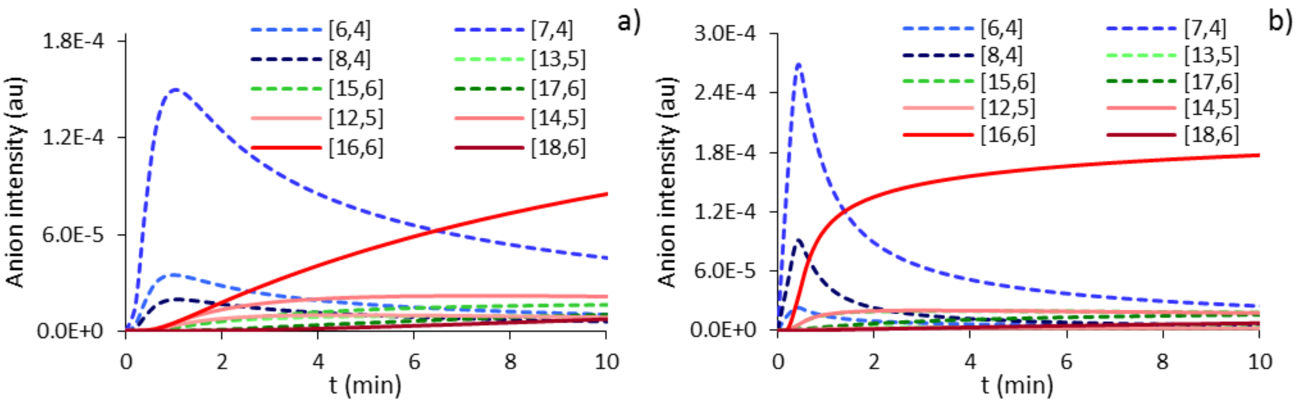

Figure S-9 – Anion intensities vs. time based on numerical simulation and anion stabilities per repeat unit.

Structures and Energies

Depicted below are the energies and entropies for all structures reported in Tables S-3 and Table S-4, where the numbering scheme is adapted from those tables. Coordinate files for all species reported in the paper are included as electronic supporting information in the form of a .xyz file.

Table S-6 – Structures, electronic, free energy, enthalpy and entropy of transition states and products

	E (a.u.)	*G* (a.u.)	*H* (a.u.)	*S* (cal mol^{-1} K^{-1})
(1) TS 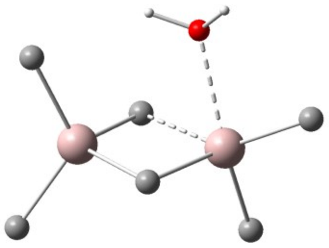	-800.733278	-800.532613	-800.471000	129.677
(1) product 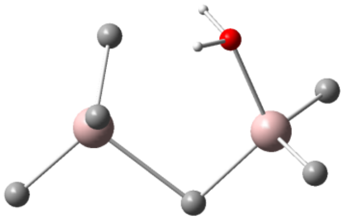	-800.750493	-800.553808	-800.487610	139.325
(2) TS 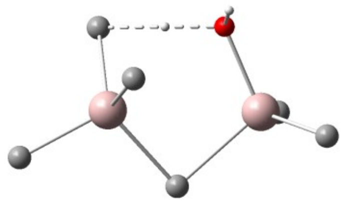	-800.741610	-800.546191	-800.483349	132.262
(2) product	-760.320530	-760.167027	-760.107914	124.413

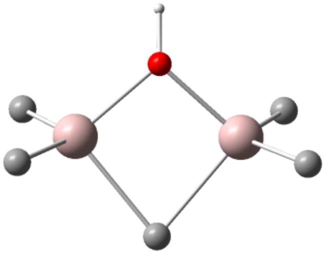				
(3) product 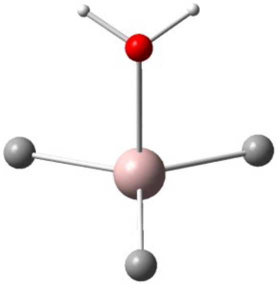	-438.593056	-438.496052	-438.448894	99.253
(4) TS 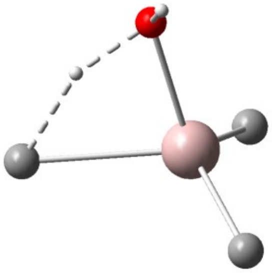	-438.567170	-438.473077	-438.427835	95.221
(4) product 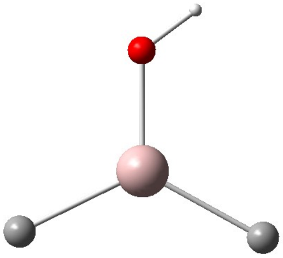	-398.119596	-398.066354	-398.025671	85.625
(5) product 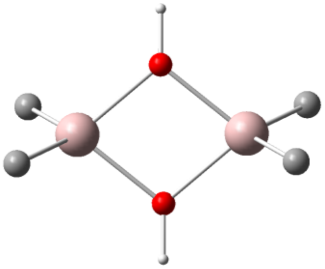	-796.336141	-796.204506	-796.145648	123.877
(6) TS	-760.270982	-760.125891	-760.063865	130.545

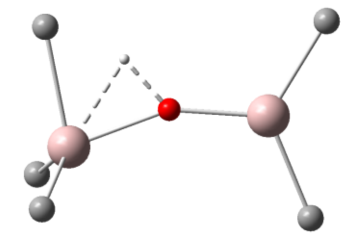				
(6) product 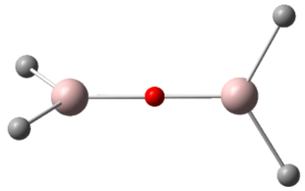	-719.825033	-719.721426	-719.662701	123.597
(7) TS 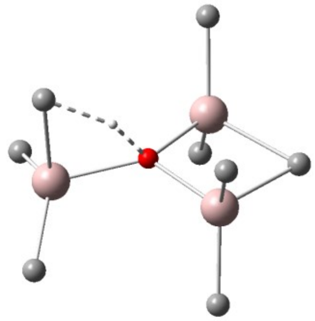	-1122.467969	-1122.219473	-1122.141181	164.779
(7) product 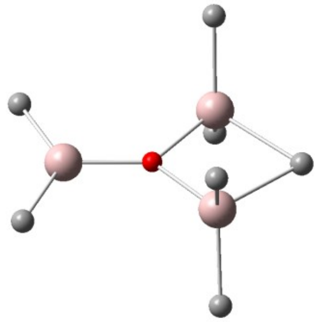	-1082.027346	-1081.821890	-1081.745947	159.835
(8) TS 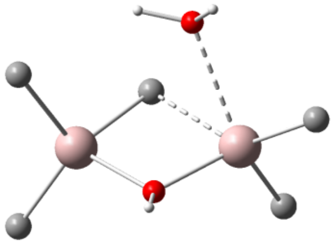	-836.743364	-836.565986	-836.503934	130.600
(8) product	-836.771368	-836.596213	-836.531180	136.873

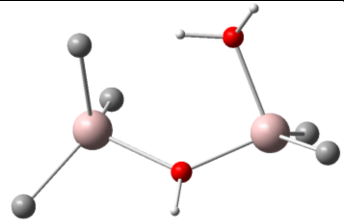				
(9) TS 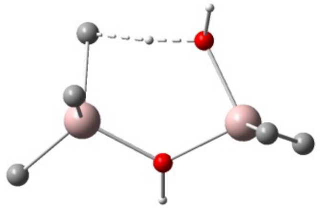	-836.768674	-836.596133	-836.532865	133.159
(9) = (5) product				
(10) product 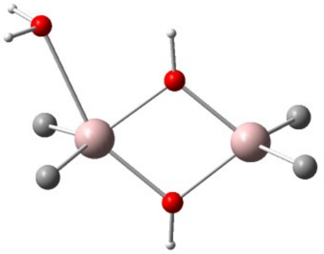	-872.775197	-872.622113	-872.556910	137.231
(11) TS 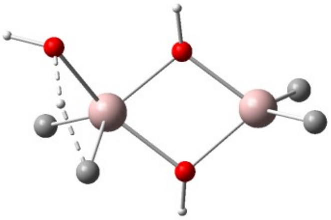	-872.747389	-872.594945	-872.533780	128.733
(11) product 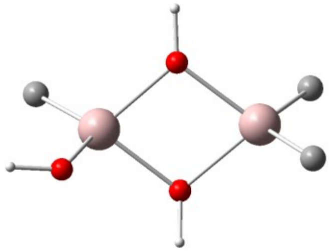	-832.315465	-832.204126	-832.147422	119.344
(12) product 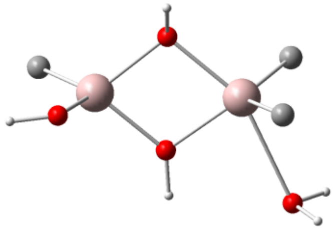	-908.754875	-908.622310	-908.559055	133.132

(13) TS 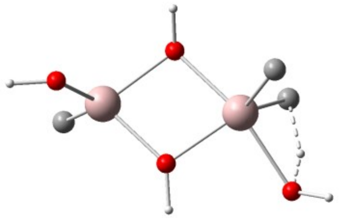	-908.726821	-908.594960	-908.535649	124.831
(13) product 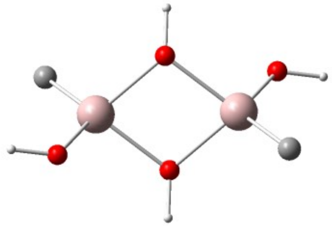	-868.294576	-868.203896	-868.149033	115.468
(14) product 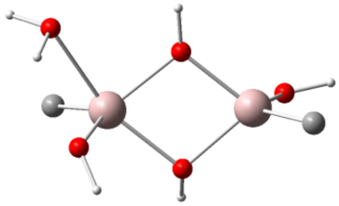	-944.742931	-944.627970	-944.568857	124.414
(15) TS 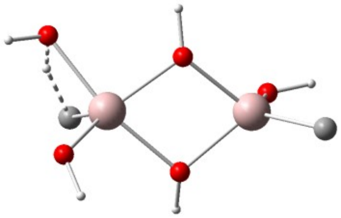	-944.708574	-944.596439	-944.539723	119.369
(15) product 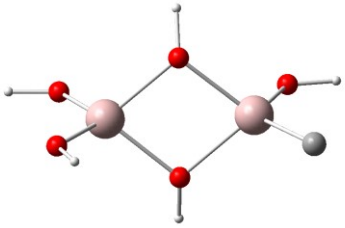	-904.270261	-904.201795	-904.147639	113.981
(16) product 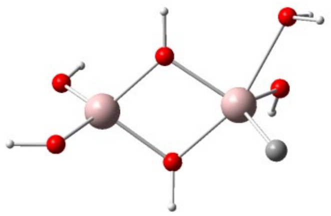	-980.720003	-980.626113	-980.568583	121.082
(17) TS	-980.685157	-980.594120	-980.539012	115.985

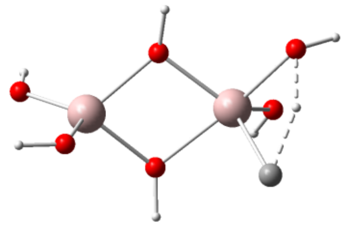				
(17) product 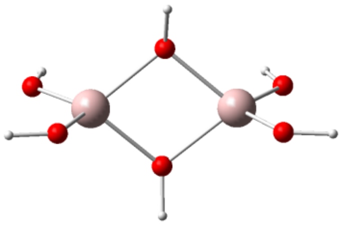	-940.246259	-940.199267	-940.146419	111.226
(18) TS 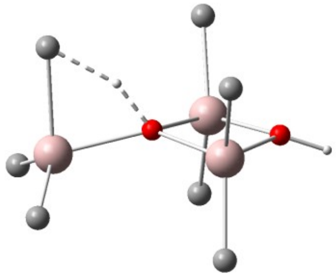	-1158.482917	-1158.255680	-1158.178333	162.790
(18) product 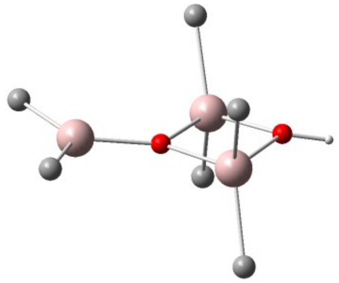	-1118.041787	-1117.856342	-1117.782521	155.369
(19) TS 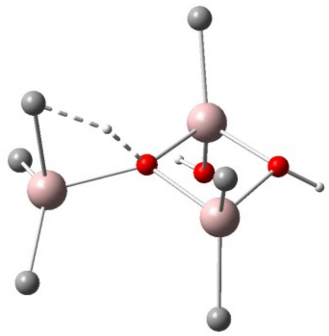	-1194.462435	-1194.256518	-1194.180332	160.348
(19) product	-1154.047275	-1153.877439	-1153.809789	142.381

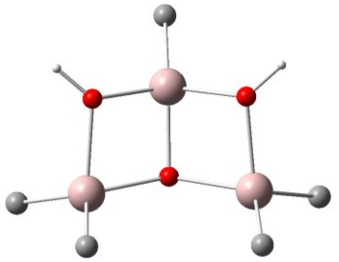				
(20) TS<br>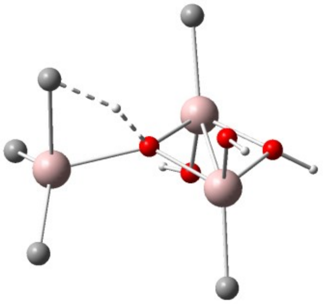	-1230.441190	-1230.256761	-1230.181837	157.692
(20) product<br>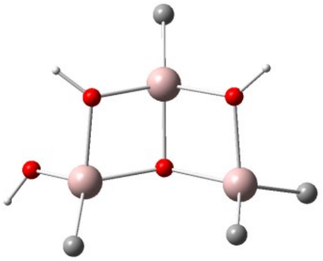	-1190.027767	-1189.878997	-1189.812747	139.435
(21) product<br>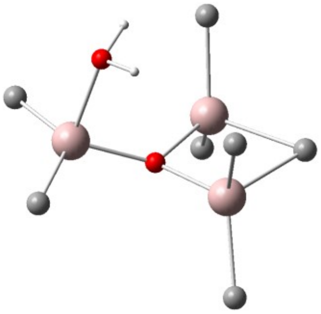	-1158.495318	-1158.262156	-1158.185775	160.756
(22) TS<br>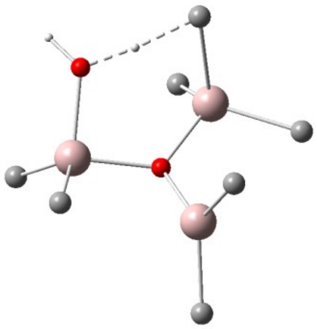	-1158.474485	-1158.248853	-1158.170289	165.353
(22) = (18) product				

(23) product 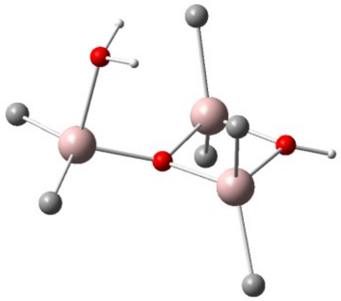	-1194.509977	-1194.297471	-1194.222558	157.667
(24) TS 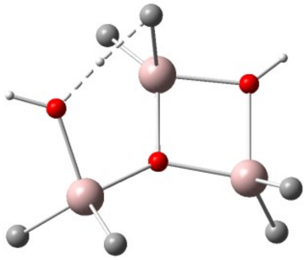	-1194.486457	-1194.279978	-1194.204374	159.122
(24) = (19) product				
(25) product 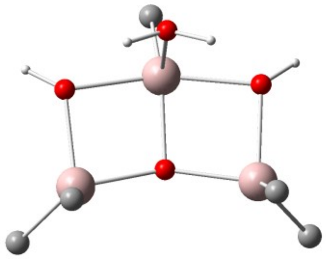	-1230.501605	-1230.310701	-1230.236181	156.841
(26) TS 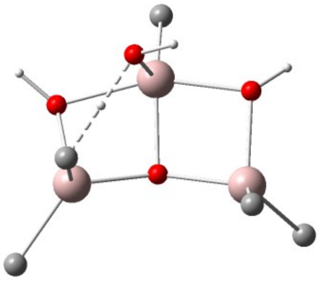	-1230.479444	-1230.289502	-1230.219151	148.067
(26) = (20) product				
(27) TS 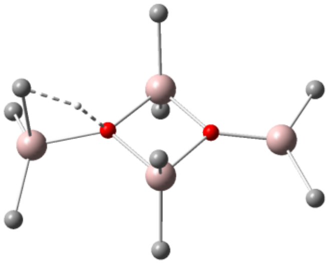	-1480.188738	-1479.908784	-1479.816445	194.343

(27) product 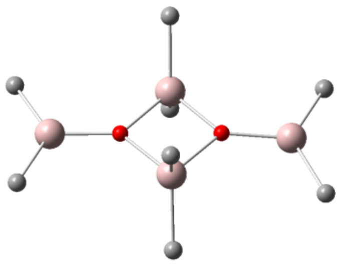	-1439.747080	-1439.510433	-1439.419059	192.313
(28) TS 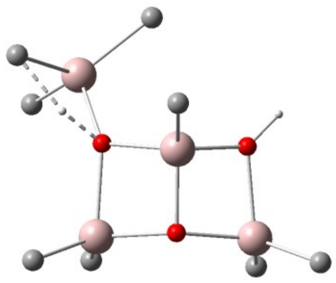	-1516.192589	-1515.929931	-1515.841242	186.661
(28) product 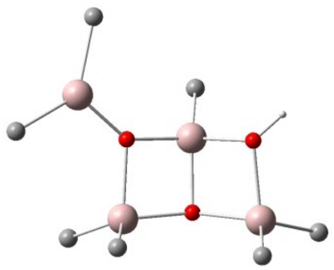	-1475.752577	-1475.530452	-1475.446335	177.039
(29) TS 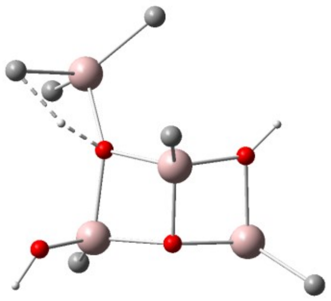	-1552.172945	-1551.931318	-1551.844256	183.239
(29) product 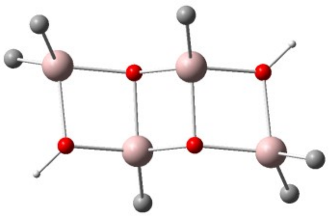	-1511.764547	-1511.557986	-1511.480217	163.679
(30) product	-1516.214955	-1515.950901	-1515.858751	193.946

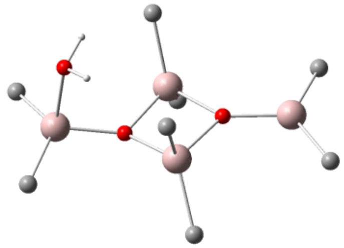				
(31) TS<br>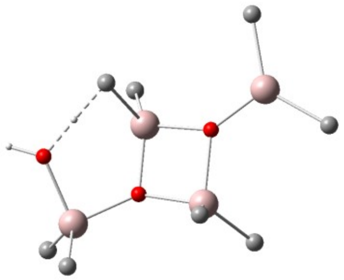	-1516.192438	-1515.932624	-1515.841488	191.812
(31) = (28) product				
(32) product<br>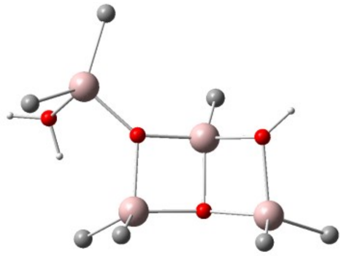	-1552.219119	-1551.972440	-1551.885035	183.960
(33) product<br>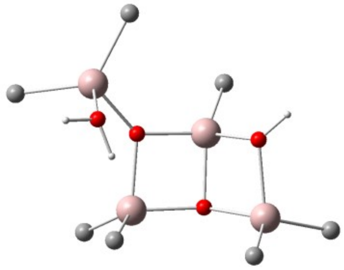	-1552.219361	-1551.972933	-1551.885185	184.680
(34) TS<br>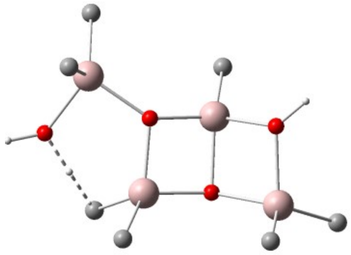	-1552.204691	-1551.960407	-1551.875601	178.491
(34) = (29) product				
(35) TS	-1552.202970	-1551.959052	-1551.873824	179.378

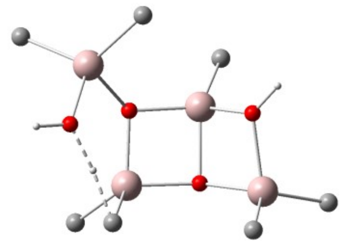				
(35) product 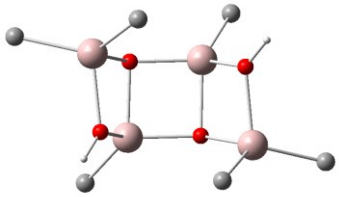	-1511.765963	-1511.561961	-1511.481965	168.366
(36) TS 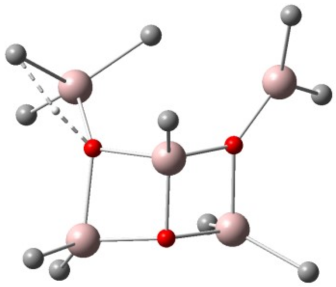	-1837.902702	-1837.582133	-1837.482494	209.708
(36) product 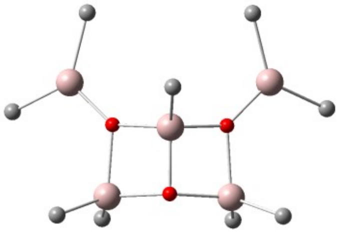	-1797.457534	-1797.182211	-1797.082513	209.832
(37) TS 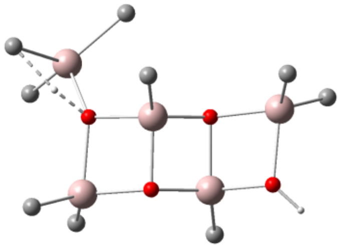	-1873.909053	-1873.609517	-1873.510751	207.870
(37) product 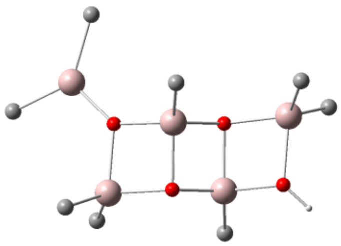	-1833.469637	-1833.211244	-1833.116529	199.345

(38) TS 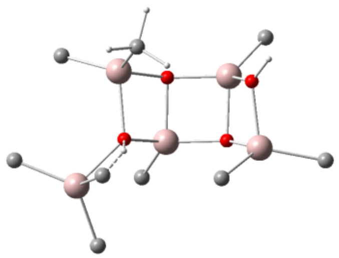	-1873.909495	-1873.608433	-1873.511474	204.066
(38) product 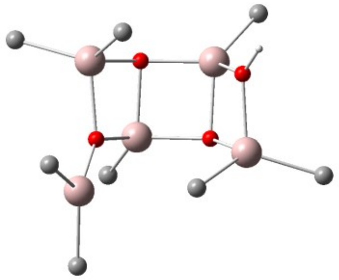	-1833.477805	-1833.216693	-1833.124803	193.398
(39) product 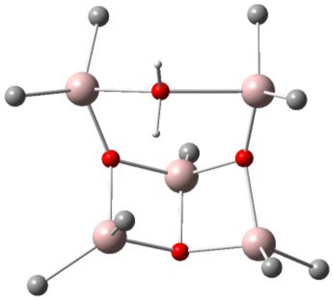	-1873.924028	-1873.617682	-1873.520693	204.130
(40) TS 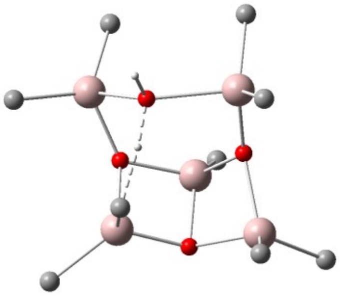	-1873.920155	-1873.614867	-1873.520855	197.864
(40) product 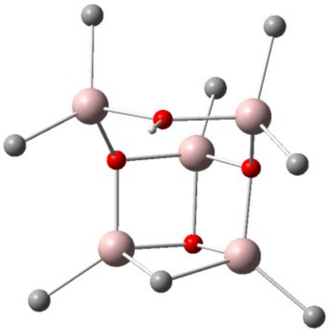	-1833.487951	-1833.225626	-1833.135230	190.256

(41) TS 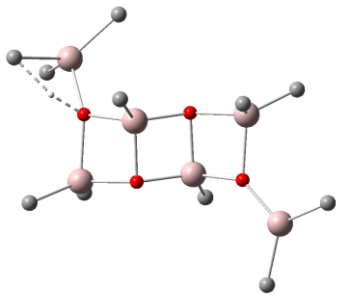	-2195.614260	-2195.262113	-2195.147180	241.898
(41) product 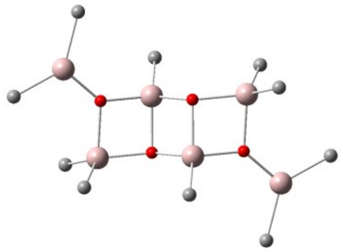	-2155.174874	-2154.863154	-2154.753057	231.720
(42) TS 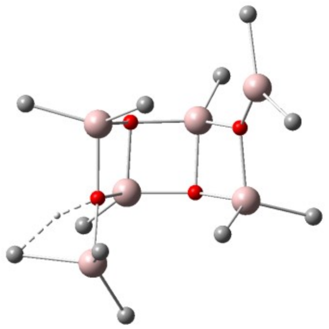	-2195.623263	-2195.265878	-2195.155996	231.266
(42) product 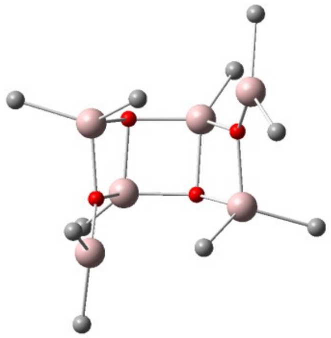	-2155.190277	-2154.873445	-2154.768532	220.808
(43) TS	-2195.632174	-2195.269658	-2195.164823	220.643

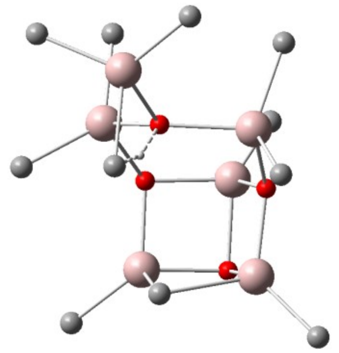				
(43) product 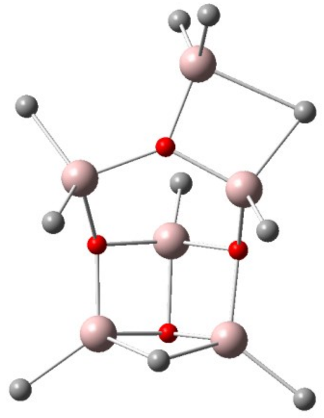	-2155.199021	-2154.877647	-2154.777351	211.090
(44) product 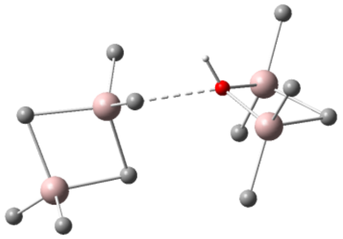	-1484.643989	-1484.294273	-1484.194175	210.674
(45) TS 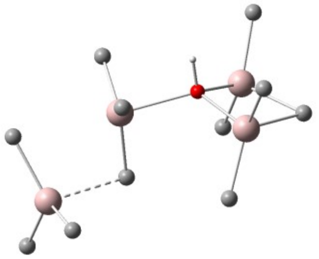	-1484.635085	-1484.282004	-1484.185671	202.750
(45) product	-1122.485107	-1122.231574	-1122.153316	164.707

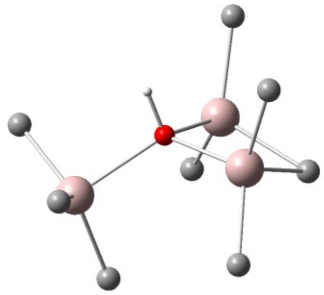				
(46) product<br>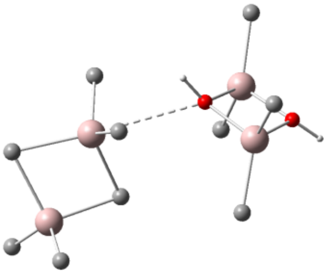	-1520.659888	-1520.330439	-1520.231916	207.359
(47) TS<br>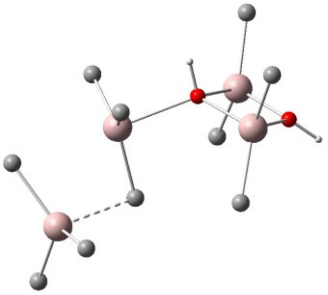	-1520.651483	-1520.318657	-1520.224016	199.189
(47) product<br>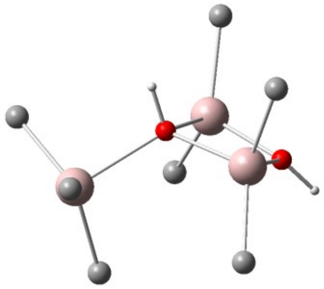	-1158.501119	-1158.268897	-1158.191523	162.848
(48) TS<br>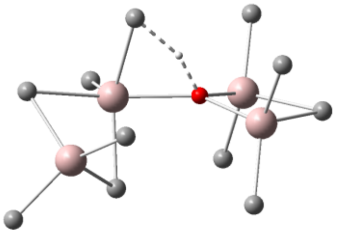	-1484.614509	-1484.261864	-1484.168796	195.878
(48) product	-1444.202106	-1443.893764	-1443.801920	193.302

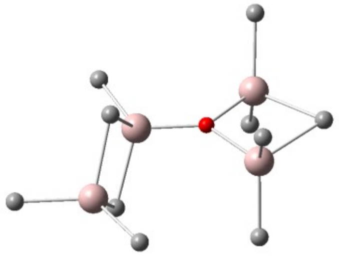				
(49) TS 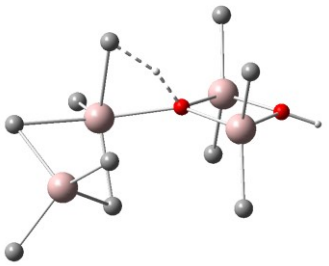	-1520.628563	-1520.298449	-1520.205085	196.501
(49) product 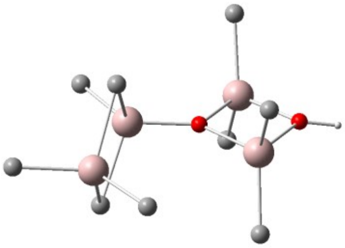	-1480.215915	-1479.930587	-1479.838024	194.814
(50) TS 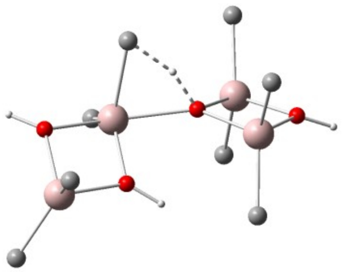	-1592.665338	-1592.379251	-1592.286639	194.920
(50) product 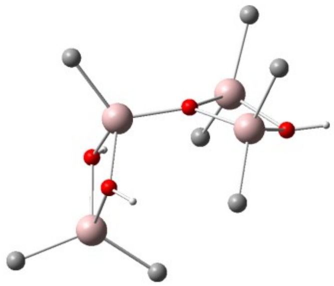	-1552.240243	-1551.992212	-1551.906495	180.408
(51) TS	-1552.203922	-1551.959011	-1551.874828	177.177

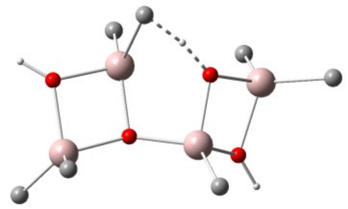				
(51) = (29) product				
(52) TS 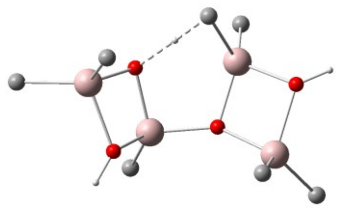	-1552.197308	-1551.953386	-1551.867918	179.883
(52) = (35) product				
(53) TS 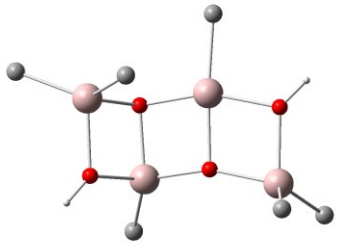	-1511.739593	-1511.533964	-1511.456636	162.749
(53) = (35) product				
(54) TS 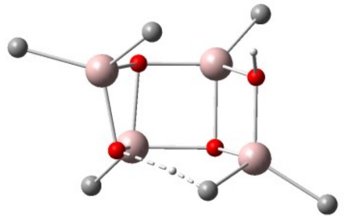	-1511.713678	-1511.507434	-1511.433245	156.143
(54) product 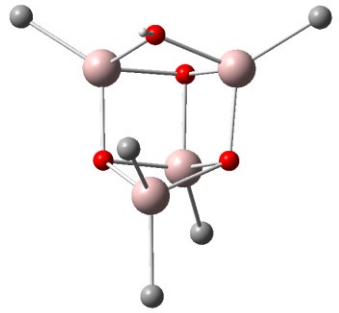	-1471.276727	-1471.109773	-1471.042959	140.622
(55) TS	-1471.240712	-1471.077622	-1471.010340	141.607

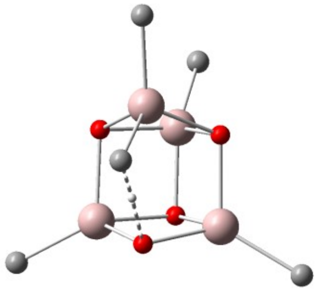				
(55) product 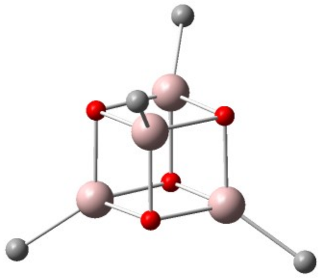	-1430.796045	-1430.670982	-1430.610913	126.428
(56) TS 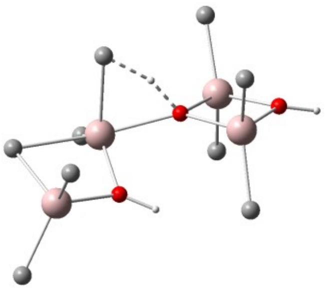	-1556.646582	-1556.338328	-1556.245683	194.987
(56) product 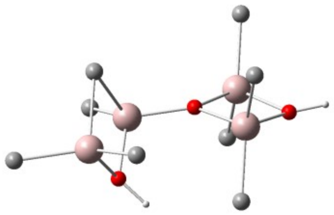	-1516.226939	-1515.960176	-1515.871359	186.931
(57) TS 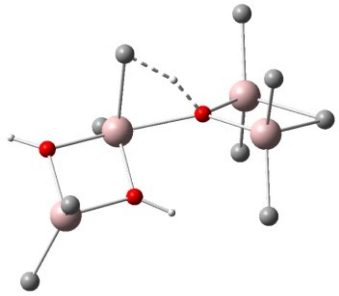	-1556.651320	-1556.343856	-1556.250490	196.506
(57) product	-1516.228779	-1515.961397	-1515.872911	186.236

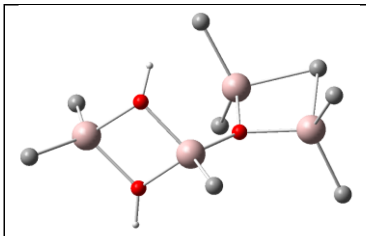				
(58) TS 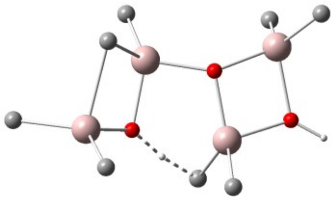	-1516.191307	-1515.926530	-1515.840261	181.569
(58) = (28) product				
(59) TS 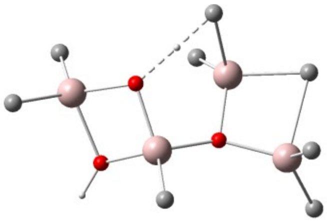	-1516.187459	-1515.922534	-1515.835859	182.423
(59) = (28) product				
(60) TS 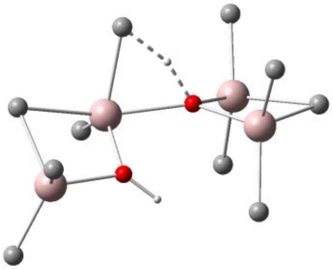	-1520.631153	-1520.303286	-1520.208197	200.132
(60) product 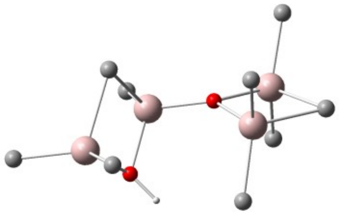	-1480.211820	-1479.924624	-1479.834198	190.318
(61) TS 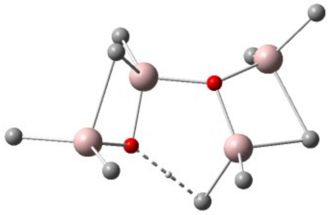	-1480.176681	-1479.891799	-1479.803423	186.004

(61) product 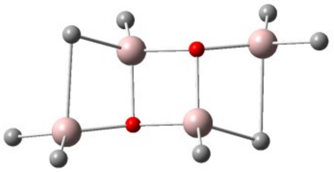	-1439.739746	-1439.494778	-1439.411601	175.061
(62) product 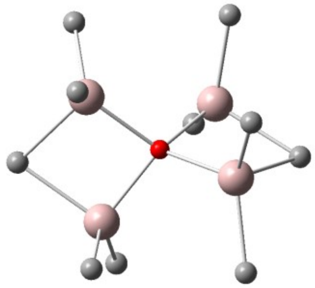	-1444.212552	-1443.895653	-1443.811354	177.422
(63) product 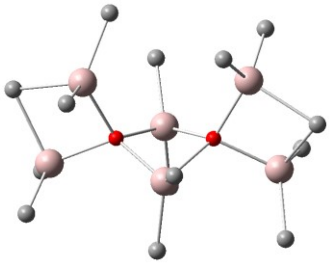	-2164.115752	-2163.658308	-2163.548358	231.409
(64) product 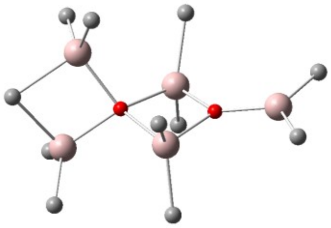	-1801.932481	-1801.583733	-1801.484837	208.145
(65) product 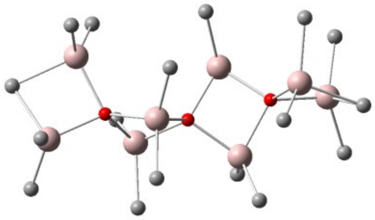	-2884.017856	-2883.418267	-2883.283753	283.108
(66) TS	-2883.996514	-2883.396257	-2883.263732	278.922

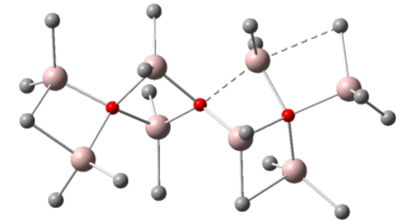				
(66) product 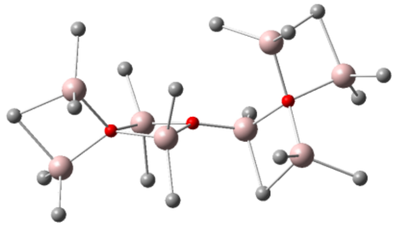	-2884.010440	-2883.413344	-2883.277653	285.586
(67) product 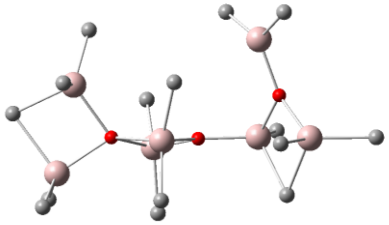	-2521.833659	-2521.349712	-2521.221149	270.584
(68) product 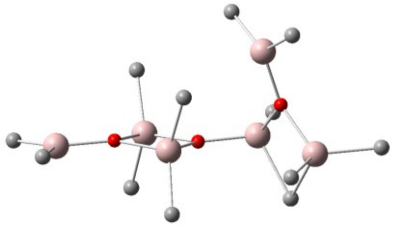	-2159.647454	-2159.275563	-2159.154375	255.062
(69) product 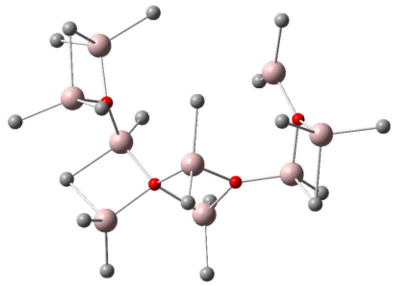	-3241.728357	-3241.105727	-3240.950418	326.875
(70) TS	-3241.711962	-3241.086688	-3240.934557	320.185

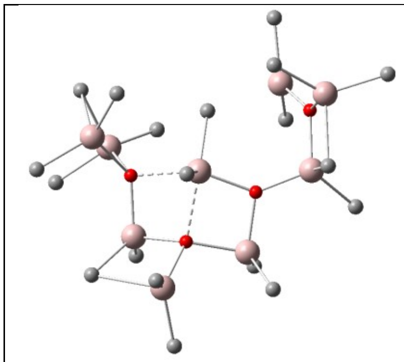				
(70) product<br>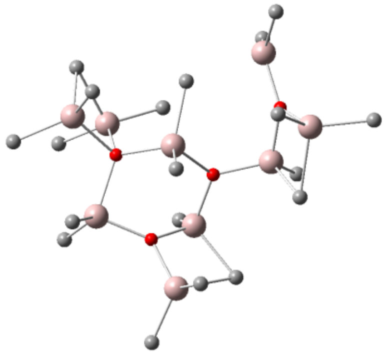	-3241.727683	-3241.099081	-3240.948888	316.108
(71) product<br>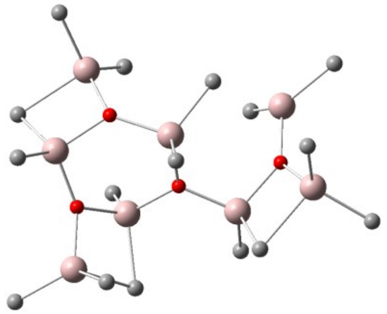	-2879.561303	-2879.041134	-2878.902610	291.548
(72) TS<br>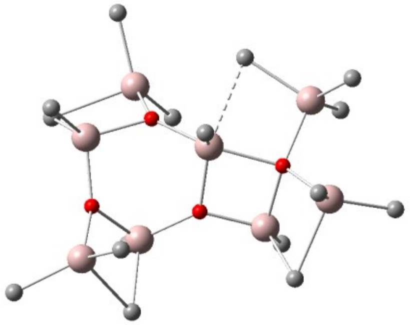	-2879.547192	-2879.026122	-2878.889443	287.664
(72) product	-2879.567115	-2879.043092	-2878.907206	285.996

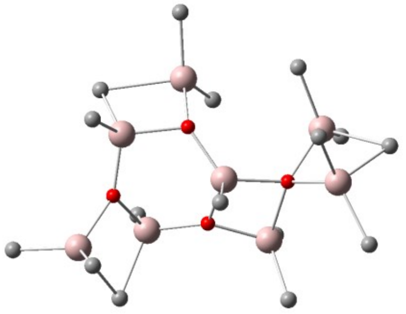				
(73) product 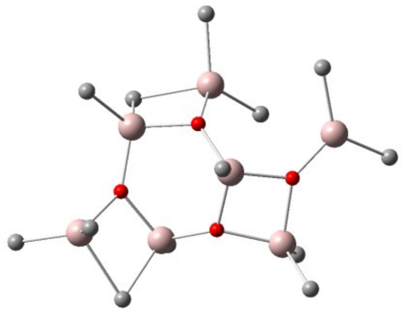	-2517.388569	-2516.971407	-2516.848464	258.756
(74) TS 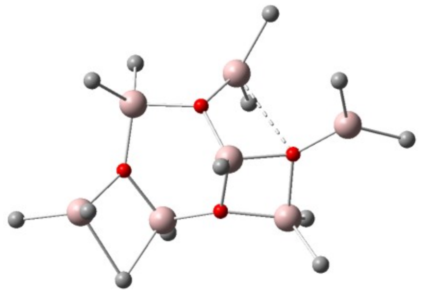	-2517.376602	-2516.961682	-2516.837435	261.500
(74) product 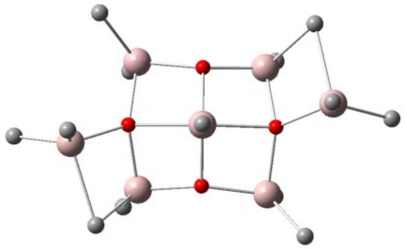	-2517.401203	-2516.981774	-2516.861197	253.775
(75) product 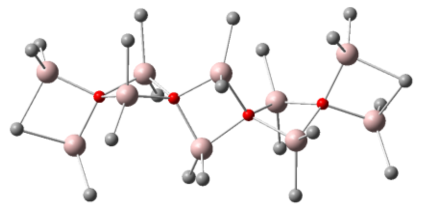	-3603.920041	-3603.180151	-3603.019631	337.843
(76) product	-3241.738059	-3241.106775	-3240.957576	314.014

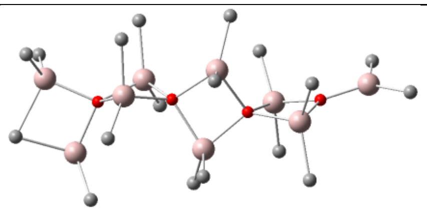				
(77) TS 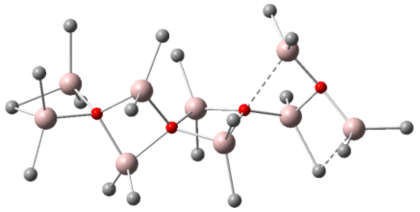	-3241.726821	-3241.094190	-3240.947888	307.919
(77) product 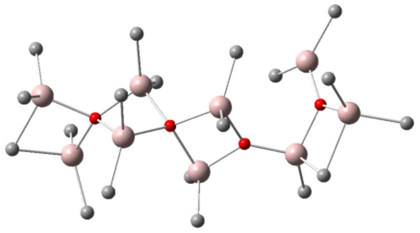	-3241.737312	-3241.110010	-3240.958277	319.350
(78) TS 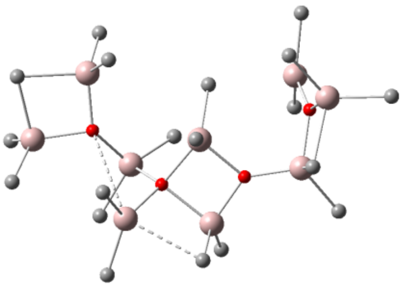	-3241.714855	-3241.091243	-3240.937705	323.148
(78) = (69) product				
